# Metabolomics and integrated network pharmacology analysis reveal SNKAF decoction suppresses cell proliferation and induced cell apoptisis in hepatocellular carcinoma via PI3K/Akt/P53/FoxO signaling axis

**DOI:** 10.1186/s13020-022-00628-1

**Published:** 2022-06-20

**Authors:** Wei Guo, Xiaohui Yao, Siyuan Lan, Chi Zhang, Hanhan Li, Zhuangzhong Chen, Ling Yu, Guanxian Liu, Yuan Lin, Shan Liu, Hanrui Chen

**Affiliations:** 1 The First Affiliated Hospital of Guangzhou University of Chinese Medicine, Guangzhou University of Chinese Medicine, Guangzhou, 510006 Guangdong People’s Republic of China; 2 The Research Center for Integrative Medicine, School of Basic Medical Sciences, Guangzhou University of Chinese Medicine, Guangzhou, 510006 Guangdong People’s Republic of China; 3 Department of Nephrology, Huizhou Municipal Central Hospital, Huizhou, 510006 Guangdong People’s Republic of China; 4 Department of Pathology, The First Affiliated Hospital of Sun Yat Sen University, Guangzhou, 510080 Guangdong People’s Republic of China

**Keywords:** Hepatocellular carcinoma, Network pharmacology, Metabolomic analysis, SNKAF decoction, PI3K/Akt/P53/FoxO signaling pathway axis

## Abstract

**Background:**

There is no comprehensive treatment method for hepatocellular carcinoma (HCC); hence, research and development are still focused on systemic therapies, including drugs. Sinikangai fang (SNKAF) decoction, a classic Chinese herbal prescription, has been widely used to treat liver cancer. However, there is no research on its core active component and target.

**Methods:**

Mouse models were established to measure the anticancer effect of SNKAF decoction on HCC. Further, we investigated the effect of SNKAF decoction on inhibition of hepatoma cells proliferation using cell viability, cloning and invasion assays in vitro. The components of SNKAF were collected from the Traditional Chinese Medicine Systems Pharmacology (TCMSP) database and TCM@Taiwan database. Metabolomic analysis was used to identify the potential genes and pathways in HCC treated with SNKAF decoction. Then, the expression of phosphoinositide 3-kinase (PI3K), Akt, P53, FoxO proteins of the potential signal pathways were detected using Western blot.

**Results:**

The animal experiments showed that SNKAF decoction inhibited tumor growth (*P* < 0.05) and induced no weight loss in the mice. In vitro data showed that HCCLM3 and MHCC97H cell proliferation was inhibited by SNKAF serum in a time- and concentration dependent manner. Further combined analysis network pharmacology with metabonomics showed that 217 target genes overlapped. The core target genes included BCL2, MCL1, Myc, PTEN, gsk3b, CASP9, CREB1, MDM2, pt53 and CCND1. Cancer-associated pathways were largely involved in SNKAF mechanisms, including P53, FoxO, and PI3K/Akt signaling pathways, which are closely related to induced-tumor cell apoptosis. In addition, Western bolt verified that 10% SNKAF serum significantly affected the main proteins of PI3K/Akt/P53/FoxO signaling pathway in both cell lines.

**Conclusion:**

SNKAF decoction-containing serum inhibited HCCLM3 and MHCC97H cell proliferation, migration, invasion, and induced-tumor cell apoptosis in-vivo. We confirmed that SNKAF decoction is a promising alternative treatments for HCC patients.

**Supplementary Information:**

The online version contains supplementary material available at 10.1186/s13020-022-00628-1.

## Introduction

Hepatocellular Carcinoma (HCC), the main form of primary malignancy of the liver, mostly originates from hepatocytes and accounts for 90% of liver cancers, with 72% of cases occurring in Asia and more than 50% in China [1]. The complicated process of liver cancer formation makes it difficult to detect HCC at an early stage using current diagnostic methods [2]. As an invasive disease often typified by the diagnosis, the prognosis of HCC is still very poor, with median overall survival (OS) of 5 months varying from 0 to 13 months [3]. As medical techniques were developed, the survival rate of patients with HCC increased generally, especially in Asian population [4, 5]. HCC is insidious and difficult to diagnose. The main curative treatment includes orthotopic liver transplantation (OLT) and surgical liver resections (LR), but only about 15% of patients are suitable for surgical resection [6]. In addition, the systemic chemotherapy is another effective treatment method [7–9]. However, most synthesized chemotherapeutic agents have serious adverse effects [10].

Given that there is no comprehensive treatment method for HCC, research and development (R&D) still focused on the systemic drug therapies. As use of alternative medicine increases, new measures for post HCC treatment are being gradually considered, particularly in developing countries, where patient consider cheap and available Chinese herbal medicines as a significant option [11].

Specifically, Traditional Chinese Medicine (TCM) has been extensively adopted as a pool filed with numerous new therapeutic agents due to its beneficial effects, accessible availability, and restricted adverse effects. Notably, it has found that Chinese medicinal formulas (CMFs) could be beneficial to HCC patients with approximately 1–2-years survival time [12, 13].

Sinikangai fang (SNKAF), prescribed traditionally for preventing and treating cancers, putatively works by decreasing the toxicity of radiotherapy and chemotherapy in tumor treatment and enhancing immunity in patients. It has been prescribed in the clinic of the first affiliated hospital of Guangzhou University of TCM to prevent and treat liver cancer. The SNKAF formula is mainly composed of *Hedyotis diffusa* Willd (Bai Hua She She Cao), *Eupolyphaga sinensis* Walker (Tu Bie Chong), *Scutellaria barbata* D. Don (Ban Zhi Lian), *Solanum nigrum* L. (Long Kui), *Akebia quinata* (Ba Yue Zha), *Bupleurum chinense* DC. (Chai Hu), *Paeonia lactiflora* Pall. (Bai shao), *Citrus aurantium* L. (Zhi Shi), *Glycyrrhiza uralensis* Fisch. (Gan Cao), *Codonopsis pilosula* (Franch.) Nannf. (Dang Sheng), *Atractylodes macrocephala* Koidz. (Bai Zhu), *Coix lacryma-jobi* Lvar. Mayuen (Roman.) Stapf (Yi Yi Ren), *Poria cocos* (Schw.) Wolf (Fu Ling), *Prunus persica* (L.) Batsch (Tao Ren), *Cremastra appendiculata* (D. Don) Makino (Shan Ci Gu). Cancer development via cell cycle arrest, apoptosis induction, and immune control can be inhibited by the major compounds in the formula [14–28]. Nevertheless, due to the multi-target and multi-substance nature of this formula, it is diffcult to investigate its underlying mechanisms. The advent of genomics, proteomics, transcriptomics, metabolomics, and serum pharmacokinetics [29–31] coincides with the research on TCM decoction, which involves multidisciplinary cooperation and complicated analytical steps [32]. It includes drug-target interaction networks and recognition of the key molecules and related pathways. Besides, the increase in effectiveness is realized by cross-referencing with a disease target database to illustrate how formulae act on core targets and affect the progression and symptoms of disease [33, 34].

Therefore, the anti-cancer effects of SNKAF on HCC and its mechanisms in vitro and vivo were assessed. Furthermore, to understand the therapeutic function of SNKAF, the principles of tumor metabolism and biological physiology were adopted to examine its anti-proliferation and enhanced-cancer cell apoptosis effects in HCC. According to the research, the integration strategy of network biology and multidirectional pharmacology is conducive to expanding the available drug target space, especially in R&D of CMF.

## Methods

### SNKAF preparation

The proportion of all medicines in SNKAF is displayed in Table 1. Based on the traditional decoction approach, all herbs were first soaked in distilled water for 30 min, heated to 100 °C, decocted twice, then filtered to eliminate the dregs with gauze. The concentrated solution was kept at 4 °C before combination. (All medicines were from the KangMei Pharmaceutical Co., Ltd, Guang zhou, China, and recognized in the TCM pharmacy of the First Affiliated Hospital of Guangzhou University of TCM).

**Table 1:** The constituents of SNKAF

Num.	Common name	Latin name	Chinese name	Weight (g)
1	Hedyotis diffusae herba	/	Bai Hua she she cao	30
2	Eupolyphaga steleophaga	Eupolyphaga sinensis Walker	tu bie chong	6
3	Scutellariae barbatae herba	Scutellaria barbata D. Don	ban zhi lian	30
4	Solanum nigrum L	/	long kui	30
5	Akebia quinata	/	Ba Yue Zha	15
6	Bupleuri radix	*Bupleurum chinense* DC	Chai Hu	15
7	Paeoniae radix alba	*Paeonia lactiflora* Pall	Bai shao	15
8	Aurantii fructus immaturus	*Citrus aurantium* L	zhishi	15
9	Glycyrrhizae radix et rhizoma	*Glycyrrhiza uralensis* Fisch	gancao	6
10	Codonopsis radix	*Codonopsis pilosula* (Franch.) Nannf	dangsheng	15
11	Atractylodis macrocephalae rhizoma	*Atractylodes macrocephala* Koidz	Bai Zhu	15
12	Coicis semen	*Coix lacryma-jobi* L var. mayuen(Roman.) Stapf	yiyiren	30
13	Poria cocos	*Poria cocos* (Schw.)Wolf	fu ling	25
14	Persicae Semen	*Prunus persica* (L.) Batsch	taoren	10
15	Cremastrae pseudobulbus Pleiones pseudobulbus	*Cremastra appendiculata* (D. Don)Makino	shancigu	15

### SNKAF serum

Guangdong Medical Experimental Animal Center provided healthy adult Sprague–Dawley (SD) rats (weight, 150–170 g), which were distributed into control group and SNKAF group (n = 15). Control group was provided with a gavage of saline, whereas SNKAF group received SNKAF decoction. According to the pharmacological experimental methodology (4th Edition), the equal dose proportion converted by body surface area between human (70 kg) and mouse (0.020 kg) is 0.018. The adult human dose of 272 g/70 kg was transformed into the rat dose (272 g × 0.018/0.16 kg = 30.6 g/kg). The rats of group B were treated with SNKAF at a dose of 5.53 g/kg. The rats were dosed twice a day for 7 days. After taking blood from the aorta 2 h after the last gavage, the serum was isolated and passed through a 0.22 um filter, aseptically aliquoted and kept at − 20 °C for later application. The animal experiments were approved by the Animal Ethics Committee at Guangzhou University of Chinese Medicine.

### HCC Xenotransplantation model

Guangdong Medical Laboratory Animal Center offered 4-week-old BALB/c nude mice. MHCC-97H cells at a density of 2 × 106/ul were mixed with Matrigel (Corning, New York, USA), and were injected subcutaneously into the right front leg of nude mice. When the tumor size was 80–100 mm3, the mice were assigned into five groups (n = 6) randomly, and treated with saline (model group), SNKAF lower dosage (15.3 g/kg), SNKAF medium dosage (30.6 g/kg), SNKAF high dosage (61.2 g/kg) and Sorafenib respectively. Sorafenib received intraperitoneal injection at 20 mg/kg every other day, and SNKAF was provided with oral gavage at different dosages every day for 15 days. The mice were examined every 2 days, and all mice were sacrificed by CO_2_ quickly without suffering, and the dissection of tumours was made at the endpoint. The Animal Ethics Committee at Guangzhou University of Chinese Medicine approved all the animal experiments.

### Hematoxylin & Eosin (H&E) staining and immunohistochemistry

Paraffin-embedded formalin-fixed tumor tissue sections were for immunohistochemistry analysis. Next, the treatment of slices with xylene and various concentrations of ethanol was done gradually, followed by immersion in distilled water. Tissue lesions were identified with H&E staining. For immunohistochemistry analysis, the slides were treated with 3% hydrogen peroxide to block endogenous peroxidase activity and incubated with 10% goat serum. Next, the slides were blocked overnight with anti-Ki67 antibody (Abcam, Cambridge, USA) at 4 °C, and next incubated with the secondary antibody. Finally, DAB detection system (Dako A/S, Glostrup, Denmark) was adopted as chromogenic agent based on the manufacturer’s guidance.

### Cell lines and cell cultures

The human liver cancer HCCLM3 cell lines were purchased from the Shanghai Zhong Qiao Zhou Biotechnology Co.,Ltd. MHCC97H cell lines were a gift from the Institute of tropical medicine of Guangzhou University of TCM. The culture of cells was prepared at 37 °C, 5% CO, followed by saturation of humidity in Dulbecco's altered Eagle's medium (DMEM) with 10% fetal bovine serum (FBS), 100 IU/mL penicillin, and 100 IU/mL streptomycin (all Hyclone, Life Sciences, Logan, UT, USA).

### Cell viability assay and colony formation assay

HCCLM3 and MHCC97H cells viabilities were examined using Cell Counting Kit 8 (CCK-8) assays. Cells were placed in 96-well plates at a density of 5000 cells/well, and adherence was achieved with incubation overnight. Cells were treated with various concentrations of SNKAF serum or Sorafenib for 72 h. CCK-8 assays. (Dojindo, Kumamoto, Japan) were adopted to measure cell viabilities according to the manufacturer’s instructions. Afterwards, a microplate reader (PerkinElmer EnSpire, Singapore) was used to measure the optical density (OD) value at 450 nm.

For colony formation investigation, the cells were cultured in 6-well plates at a density of 5 × 10^3^ cells per well. After cell attachment, SNKAF serum or Sorafenib was put into the wells for 24 h, followed by cultivation with a fresh complete culture medium for another 2 weeks. The fixing of generated colonies was conducted in 4% paraformaldehyde, with subsequent staining by crystallization purple, photographing, and counting under a microscope.

### Wound healing assay and transwell invasive assay

In terms of wound healing assays, the 4 × 10^5^ cells were seeded into 6-well plates for > 90% confluency, followed by scratching with a 1-mL pipette tip. Next, the recording of cells was done at 0 and 48 h after initiating the wound healing. Image J software (National Institute of Mental Health, Bethesda, Maryland, USA) was adopted to analyze the healing distance and region. For transwell invasive assays, the 8-um transwell chambers were coated with Matrigel and then placed into 24-well plates before the experiment. Later, upper chambers with cells were added at a density of 1.5 × 10^5^ according to the suggested treatment, whereas complete DMEM medium with 10% FBS filled the lower chamber for 48 h. In the end, a cotton swab was used to erase the upper cells, and after being fixed with 4% formaldehyde, the 30-min dying of cells invaded into the bottom of chamber with 0.5% hematoxylin solution was made, next to photographing for detection.

### Flow cytometry analysis

For the drug efflux assay, the cells were placed in 6-well plates at a density of 3 × 10^5^ cells/well, followed by treatment with various doses of SNKAF serum or sorafenib for 48 h. For cell cycle exploration, the cells were fixed overnight in ice-cold 70% ethanol at 20 °C. Next, phosphate-buffered saline (PBS) was adopted to wash cells, with subsequent staining with 50 mg/mL propidium iodide (BD Biosciences, San Jose, CA, United States). For apoptosis exploration, the Annexin V-FITC Apoptosis Staining/Detection Kit (BD Biosciences) was adopted to stain the cells. All flow cytometry analyses were made with BD Accuri C5 or LSRFortessa and FlowJo software.

### Hoechst 33258 and terminal deoxynucleotidyl transferase (TdT)-mediated dUTP nick end labeling (TUNEL) staining

Immunofluorescence was used to analyze HCC apoptosis after SNKAF serum administration. The seeding of growth phase cells into 6-well plates was performed at a density of 1 × 10^5^ cells. The cells were incubated with various concentrations of SNKAF or Sorafenib. After 48-h incubation, the cells were washed with PBS and fixed by 4% polyformaldehyde. Next, the cells were washed with PBS buffer, and Hoechst 33258 solution (10 ug/mL) was used to stain the cells after washing twice. Thereafter, they were incubated in the dark for 20 min to obtain the stable samples. The InSitu Cell Death Detection Kit (Roche) was used for TUNEL analysis according to the manufacturer’s protocols. Finally, an anti-fluorescence quenching mounting solution was employed to mount cells before putting them under observation with a confocal microscope (Zeiss LSM800).

### Metabolomic profiling

After collection, the plasma samples were centrifuged for 10 min at 1000 rpm. The thawing and equilibrium of all samples were performed at 4 °C. Then, 1 mL of acetonitrile: methanol: ddH2O mixed solution (2:2:1, v/v/v) was added to the plasma samples, mixed and centrifuged at 4 °C for 10 min at 12,000 rpm. Next, 850 ~ 900 uL of supernatant were transferred and made to evaporate to dryness. Subsequently, the dissolved samples were centrifuged after being vortex-mixed with 300 uL of 2-chlorobenzalanine solution (4 ppm). Lastly, the supernatant was screened through a 0.22 um membrane to prepare samples for the LC–MS analysis.

Chromatographic conditions: the plasma samples were made using the Thermo Scientific Vanquish UHPLC-Q Exactive system and Hyperil Gold column(C18) column (100 mm × 2.1 mm i.d., 1.7 µm; Waters, Milford, USA), with the temperature of the column kept at 40 °C. Solvent A was water (including 0.1% formic acid), whereas solvent B was acetonitrile/isopropanol (1/1) (including 0.1% formic acid). The flow rate was set at 0.20 mL/min, and each sample was injected within volume of 20 ul. The gradient elution program was shown below: 0 min 2% B, 0–2 min 2% B, 2–12 min 98% B, 12–14 min 98% B, 14.0–14.1 min 2% B, 14.1–17 min 2% B.

The electrospray ionization-mass (ESI-MS^n^) spectrometry experiments were conducted using an Orbitrap Q Exactive™ HF mass spectrometer to examine the stability of the instrument. The spray voltages were both 3.2 kV for the positive and negative modes. Sheath gas flow rate were set at 10 ~ 40arb and the capillary temp at 320 °C. An HCD scan was carried out to make data dependent acquisition-MS/MS experiments, and several unnecessary data in the MS/MS spectra were eliminated with dynamic exclusion.

### Establishment of an ingredient-target-liver cancer network

The information on SNKAF decoction constituent compounds was gathered from TCM systems, and the database platform used included the TCM Systems Pharmacology Database (TCMSP, http://lsp.nwu.edu.cn/index.php), TCM ingredient database (TCMID, http://119.3.41.228:8000/), a Bioinformatics Analysis Tool for Molecular mechANism of TCM (BATMAN-TCM, http://bionet.ncpsb.org/batman-tcm/), and the TCM database@Taiwan (TCM@Taiwan, http://tcm.cmu.edu.tw/zh-tw/). The ingredients were based on the drug-likeness (DL) and oral bioavailability (OB) values, and the retaining of ingredients was realized if DOB ≥ 30%, DL ≥ 0.18, and Caco_2_ > 0, a standard indicated by the TCMSP database.

Then, the potential molecular targets of each recognized compound were identified using the Genecards database, BATMAN-TCM (https://www.genecards.org/), and the STRING database (https://string-db.org/). The ingredient–target networks were applied via Cytoscape software (version 3.2.1). The subjection of target genes to the Database for Annotation, Visualization, and Integrated Discovery (DAID, https://david.ncifcrf.gov/) and the Comparative Toxic Genetics Database (CTD, http://ctdbase.org/) was made to implement Gene Ontology (GO) and Kyoto Encyclopedia of Genes and Genomes (KEGG) enrichment for functional enrichment analysis. The clusterProfiler software package for R3.5.3 was utilized to evaluate the hidden biological functions, and *P-*value < 0.05 suggested greater enrichment [35].

### Data analysis

Principal Component Analysis (PCA) was adopted for quality control analysis. The classifications for different groups were observed with Partial Least Squares Discrimination Analysis (PLS-DA) and Orthogonal Partial Least Squares Discrimination Analysis (OPLS-DA). Ions with variable importance (VIP) > 1 and *P*-value < 0.05 were considered as differentiated metabolite ions. In the advanced data exploration, the heatmap analysis and KEGG enrichment exploration were made via the Majorbio Cloud Platform. Pathway analysis was made with Metabo Analyst.

### Statistical analysis

One-way analysis with the GraphPad Prism v6.0 software (GraphPad Software, Inc., San Diego, CA, USA) was done to compare different groups. Data were shown as the mean ± SD of at least three repeated experiments. It was thought that *P-value < 0.05, **P-value < 0.01 and ***P-value < 0.001 are statistically significant.

## Results

### SNFAF suppresses the growth of liver cancer xenotransplantation model in vivo

Although SNKAF has been empirically adopted as an adjuvant in clinically treating hepatocarcinoma, current available evidence illustrating its mechanisms is limited. The anticancer-like activity of SNKAF was assessed in nude mice with xenografted tumors. First, MHCC-97H cells were subcutaneously injected into the mice and then randomly assigned to five groups: (1) Model (normal saline); (2) SNKAF-Low; (3) SNKAF-Medium; (4) SNKAF-High and (5) Sorafenib.

**Fig. 1:**
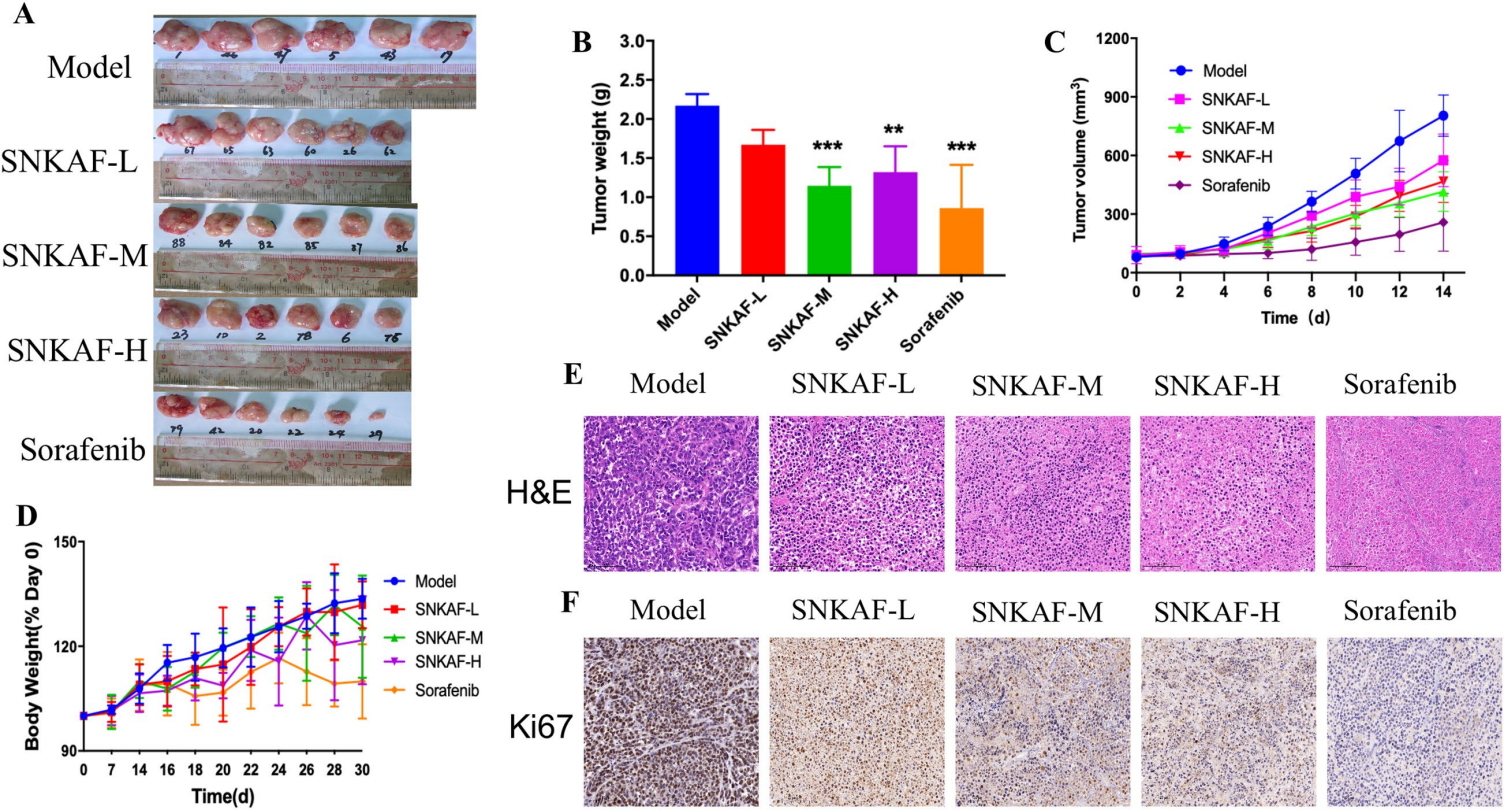
SNKAF decoction inhibited tumor growth in HMCC-97H cell tumor xenograft models. **A** The anticancer-like activity of SNKAF decoction was evaluated in nude mice with xenografted tumors. A total of 30 mice were subcutaneously injected with HMCC-97H cells and then randomly assigned to five groups: (1) model group (treated with saline), three SNKAF decoction dosage and Sorafenib groups. **B** The mice were sacrificed for tumor tissue collection, and the tumors were weighted after 15 days of administration. **C** The tumor volume was measured every 2 days. **D** The mice weight was measured every 7 days before expression and the weight was measured every other day when drug treatment. Error bars represent the mean ± SE. **E** The H&E staining, **F** IHC detection of Ki67 expressions from the indicated groups. (*P < 0.05, **P < 0.01, ***P < 0.001 v.s. model)

The mice were sacrificed for tumor tissue collection after administration of SNKAF decoction or Sorafenib. SNKAF significantly inhibited tumor volume (Fig. 1A and B), and the average tumor volume was remarkably smaller from day 6 till the end of the research in the SNKAF-High-group, SNKAF-Medium and low group showed significant inhibition from day 8 compared with the model group (Fig. 1C). During the whole experiment, SNKAF treatment induced no weight loss in the mice, however obvious toxicity was observed after Sorafenib administration (Fig. 1D). H&E pathological staining (Fig. 1E) showed that Sorafenib treatment led to extensive cell death according to smeared cell morphology and nuclear staining. In addition, treatment with SNKAF also caused cell death. Ki67 is an important cell nuclear proliferation marker [36], the tumor cell greatly impaired by the SNKAF or sorafenib treatment (Fig. 1F). Together, these findings confirmed that SNKAF inhibited tumor growth in human liver tumor-bearing xenograft mice.

### SNKAF serum exerts anti-proliferation, migrative and invasive potentia effects on HCCs

**Fig. 2:**
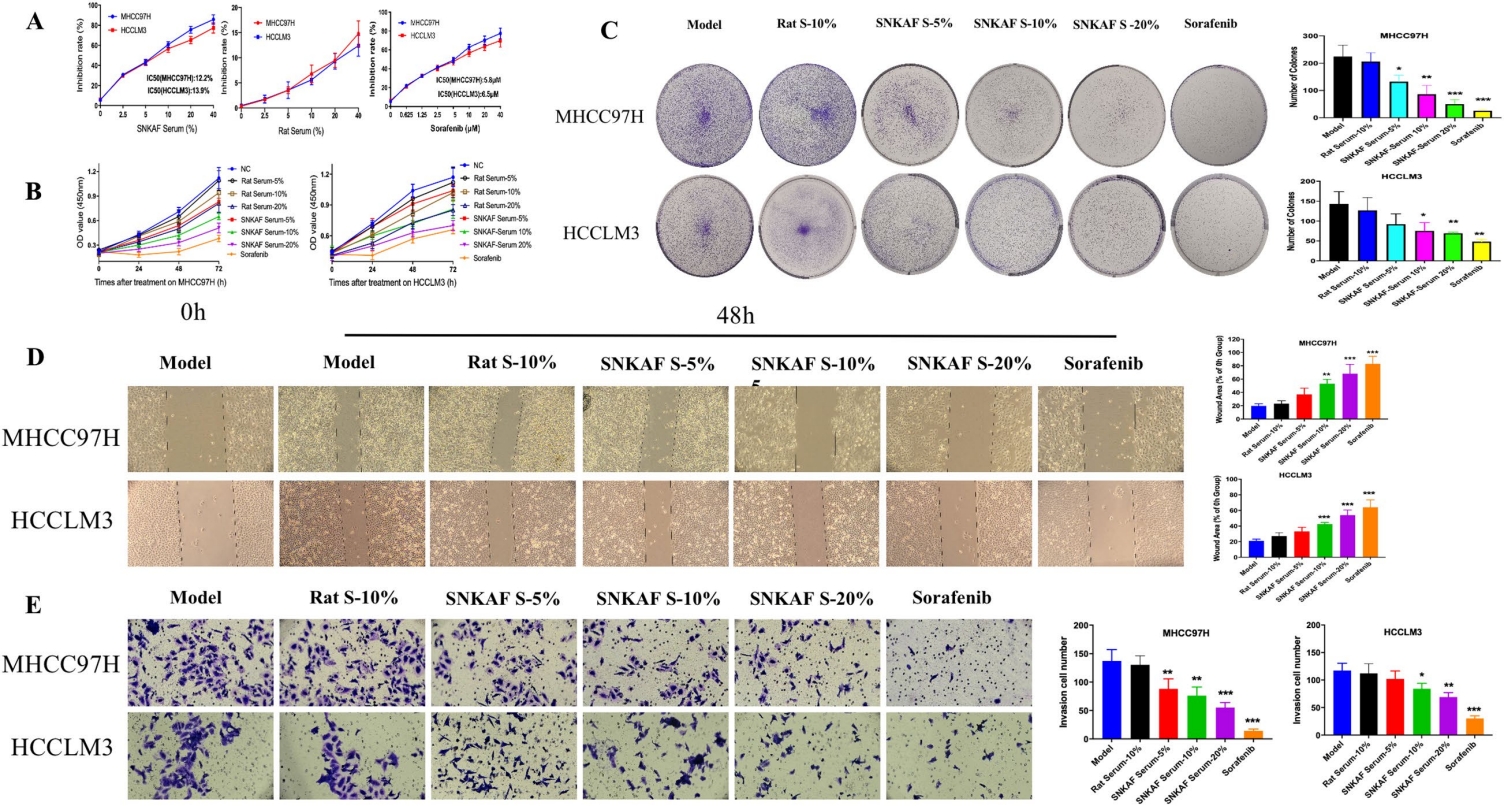
SNKAF serum inhibited the proliferation, migrative and invasive potentia of HCC cells. **A**, **B** MHCC97H and HCCLM3 cells were treated with a gradient concentration of SNKAF serum, rat blank serum and sorafenib for 48 h or certain time intervals (0, 24, 48 and 72 h) using CCK8 assays. **C** The influence of SNKAF serum on colony formation of MHCC97H and HCCLM3 cells. **D** Phase-contrast images for wound healing after administration SNKAF serum for 48 h. right: quantification of migration healing areas. **E** images (left) and quantification (right) of decreased cell number in transwell chambers with or without SNKAF serum on MHCC97H and HCCLM3 cells

SNKFA significantly inhibit liver cancer growth in vivo. The anti-cancer effect of SNKAF on cancer cell lines, including HCCLM3 and MHCC97H, was assessed. The viability of both hepatoma cell lines was inhibited in a concentration- and time-dependent manner by CCK-8 assays (Fig. 2A, B). Figure 1A showed the half-maximal inhibitory concentration (IC50) of SNKAF serum or Sorafenib needed for the suggested cell lines. To be specific, the IC50 values of SNKAF at 48 h for HCCLM3 and MHCC97H were 13.9% and 12.3%, whereas those of Sorafenib were 6.5 uM and 5.5 uM, respectively. In MHCC97H cells, the viability of the cells was inhibited in the three SNKAF serum groups compared to the blank control cultures at 48 and 72 h. Rat blank serum at 20% was observed to have a markedly inhibitory effect on cell proliferation at after 48 h. however, at a concentration of 10%, it had no effect on cells at 24–72 h compared with the blank control group. In HCCLM3 cells, SNKAF serum at concentration of 10% and 20% had an obvious inhibiting effect compared with the blank control group at 48 and 72 h. The rat blank serum at 20% could inhibit the cell growth at 24 h. In contrast, the concentration at 10% had no effect on cells at 24–72 h compared with the blank control group. Thus, the blank serum at 10% is better for conducting deeper research.

Then, a colony formation assay confirmed the inhibitory effect on HCCs. A reduced number of colonies that formed in the different groups is shown in Fig. 2C, SNKAF serum blocked the colony-forming capability in a dose-dependent manner in both HCCLM3 and MHCC97H cells. Wound healing assays were adopted to assess the potential effects of the SNKAF serum on the migrative capacity of cells. As shown in Fig. 2D, the gap widths and regions of the untreated group narrowed down more quickly than those of the SNKAF serum or Sorafenib group from 0 to 48 h. Besides, according to chamber invasive assay, the aggressive cell number was greatly decreased with SNKAF serum treatment at concentration of 5 ~ 20% (Fig. 2E). Altogether, these data suggest that SNKAF serum could suppress HCC cell proliferation, invasive and migration in vitro.

### SNKAF serum induced apoptosis in HCC Cells and HCC cell cycle arrest at the S and G2/M phase

**Fig. 3:**
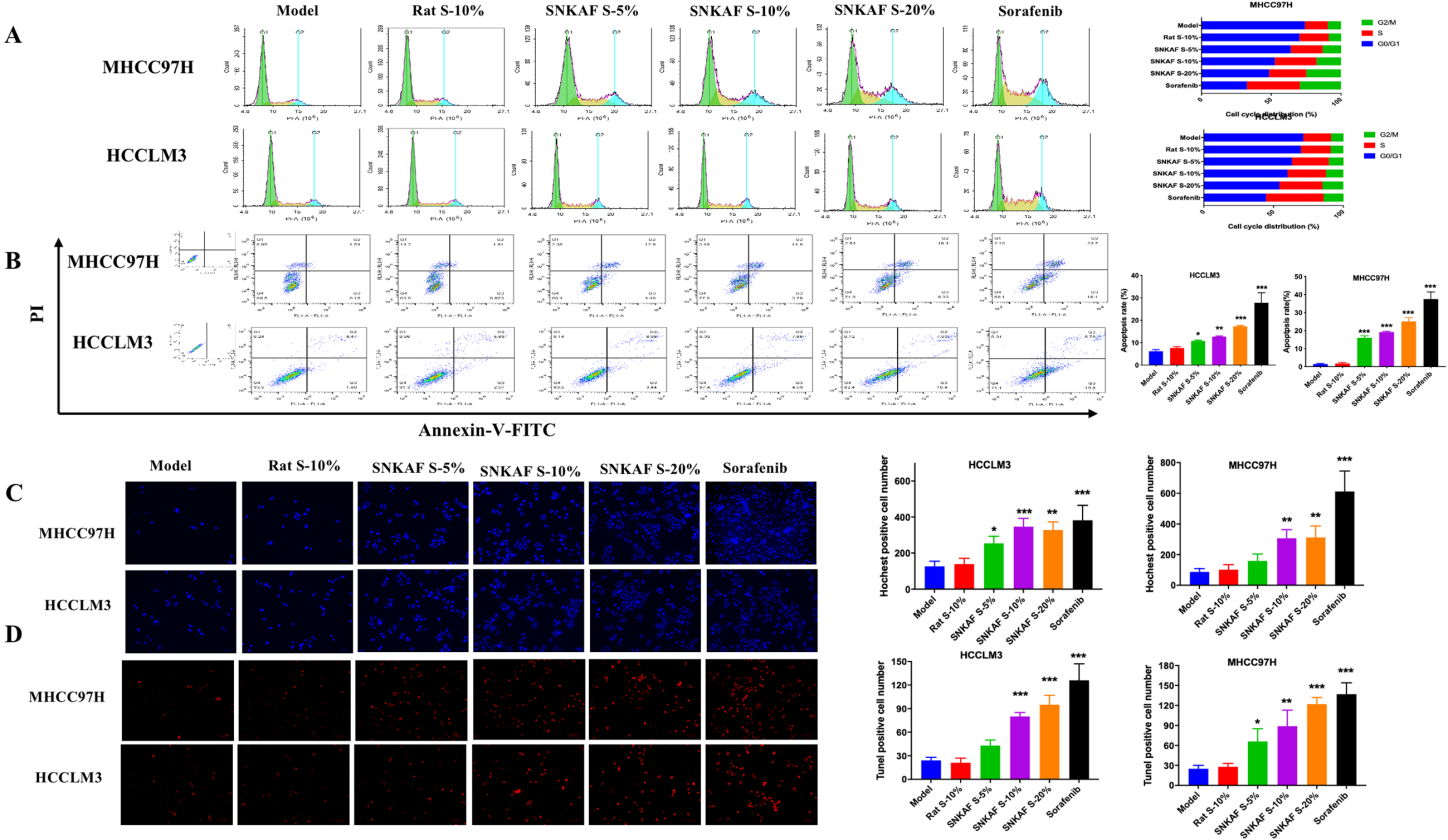
SNKAF Serum Induced apoptosis and arrested HCC cell cycle at the S and G2/M Checkpoint. Cell cycle demonstrated that different dosages of SNKAF serum relieved the G2/M and S arresting in both MHCC97H and HCCLM3 cells using flow cytometry (**A**). Apoptosis analysis of the MHCC97H and HCCLM3 cells treated with SNKAF serum, blank rat serum and sorafenib using flow cytometry (**B**). Hoechst 33258 staining (**C**) with TUNEL assay (**D**) showed typical apoptotic morphology changes in cells after indicated treatment

We investigated the anti-cancer mechanism via which SNKAF serum inhibits HCC cells proliferation. In the S phase, the cell synthesises DNA in its nucleus, duplicating a microtubule- organising structure called the centrosome to isolate DNA during the M phase. In the G2/M phase, the cell grows rapidly to produce proteins and organelles and starts reorganizing its contents in preparing for mitosis. An increase in the S phase with the G2/M population shows a rise in the apoptotic cells. Propidium iodide (PI) staining was adopted to investigate the roles of SNKAF serum in cell cycle distribution using flow cytometry analysis. As shown in Fig. 3A, the flow cytometry results revealed that SNKAF serum could induce G2/M and S checkpoints in both liver cancer cell lines. Sorafenib arrested the cell cycle at G2/M and S phase in MHCC97H cells (67.44%) and HCCLM3 cells (55.44%). SNKAF serum with different doses induced cell cycle arrest at the G2/M phase in MHCC97H cells (36.03%, 37.43%, and 51.57%) compared with the rat serum group (29.84%) and HCCLM3 cells (36.88%, 39.92%, and 45.75%) compared with 30.43% in rat serum group.

Another significant mechanism indicated that drugs induced cell death by apoptosis [37]. Thus, we detected the apoptotic events with annexin V/PI staining. As shown in Fig. 3B, the percentage of early and late apoptotic events in HCCLM3 and MHCC97H cells was about 28.56% and 39.8%, respectively, after being exposed to Sorafenib. When SNKAF serum at 5%-20% dosage was administrated, the percentage of HCCLM3 apoptotic cells grew to about 10.4%, 12.25%,and 17.43%; MHCC97H apoptotic cells increased to about 17.3%, 18.69% and 25.73%. To further confirm the protective roles of SNKAF serum in HCC cells, Hoechst staining was adopted to examine apoptotic features and TUNEL staining was used to examine DNA fragmentation and cell death in HCCs with or without SNKAF serum treatment. As shown in Fig. 3C and D, SNKAF serum greatly reduced the percentage of apoptotic cells tested by Hoechst and TUNEL staining in both HCCLM3 and MHCC97H cells. Together, these results confirmed that SNKAF serum promoted cells apoptosis in HCCs.

### Network pharmacology predicted the potential target of SNKAF on HCC

**Fig. 4:**
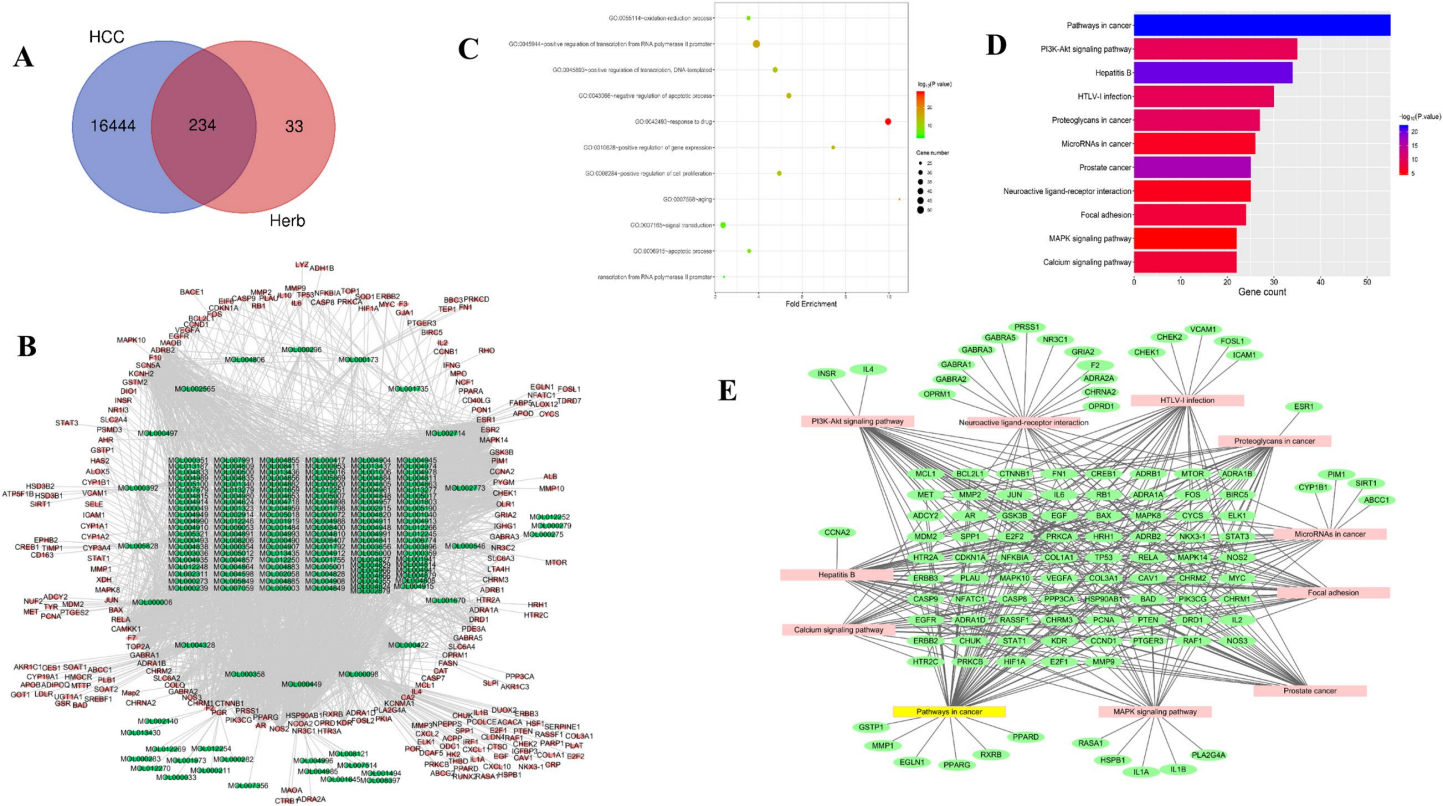
Network pharmacology analysis of SNKAF treating HCC. **A** The Venn analysis showed the crosstalk of DEGs in SNKAF and hepatocellular carcinoma; **B** PPI network of herb’s compound targets against HCC. The nodes with red borders represent the hub genes. **C** The GO enrichment analysis of potential targets by ClueGO, all data collected have a *p*-value ≤ 0.01. **D** The KEGG pathways enrichment analysis by ClueGO. All pathways have a *p*-value ≤ 0.01. **E** Gene target-pathway signal network, the green ellipse represents the different gene targets and the square colors represent the pathways

The chemical ingredients of SNKAF were collected from the TCMSP Databases. As a unique systems pharmacology platform of TCM, the database captures the associations between drugs, targets and diseases. Firstly, we screened candidate compounds in the TCMSP database for OB, Caco-2 permeability, and DL using the criterion (OB ≥ 30%, DL ≥ 0.18, and Caco-2 ≥ 0), the screening yielded 196 candidate compounds in total (Additional file 1: Table S1). Then, 2537 genes were extracted from the Genecard (Fig. 4B), of which 234 of them overlapped with the HCC targets in the Venn diagram analysis (Fig. 4A). To identify the functions and mechanisms of SNKAF, GO and KEGG pathway enrichment analysis were made based on the background of all human genes. For GO-term analysis, positive control of transcription from RNA polymerase II promoter, response to drug, signal transduction, negative control of apoptotic process, positive control of transcription, DNA-templated, positive control of cell proliferation, oxidation–reduction process, apoptotic process, positive control f gene expression, aging, negative control of transcription from RNA polymerase II promoter were most greatly related to SNKAF action (Fig. 4C). For KEGG pathway discussion, anti-cancer mechanisms of SNKAF might be closely related to various intervened pathways such as the pathways in cancer, PI3K-Akt signaling pathway, hepatitis B, proteoglycans in cancer, microRNAs in cancer, neuroactive ligand-receptor interaction, prostate cancer, focal adhesion, MAPK signaling pathway, and calcium signaling pathway (Fig. 4D). The filtering of pathway networks for the hub genes was made in Fig. 4E, the squares indicated that the pathways and the target genes taking part in the network were set by the circle.

### Metabolomics analysis identified the Differential Metabolism-target of SNKAF in plasma of Mice

**Fig. 5:**
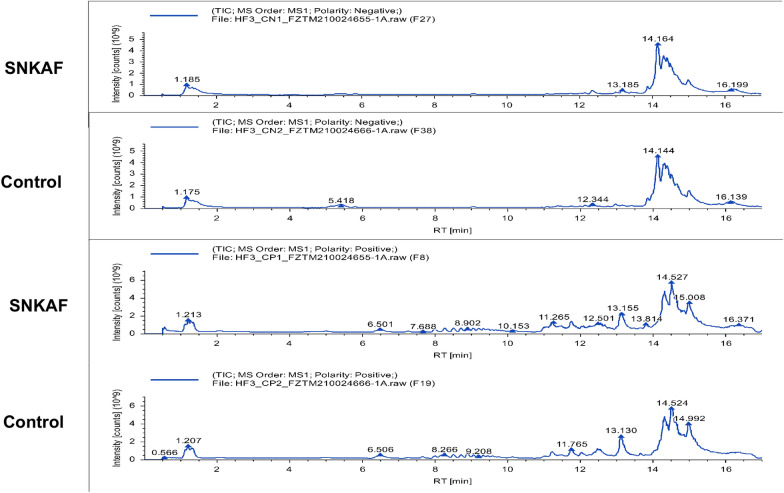
BPI chromatograms of the SNKAF serum and control groups in the positive and negative ion modes

**Fig. 6:**
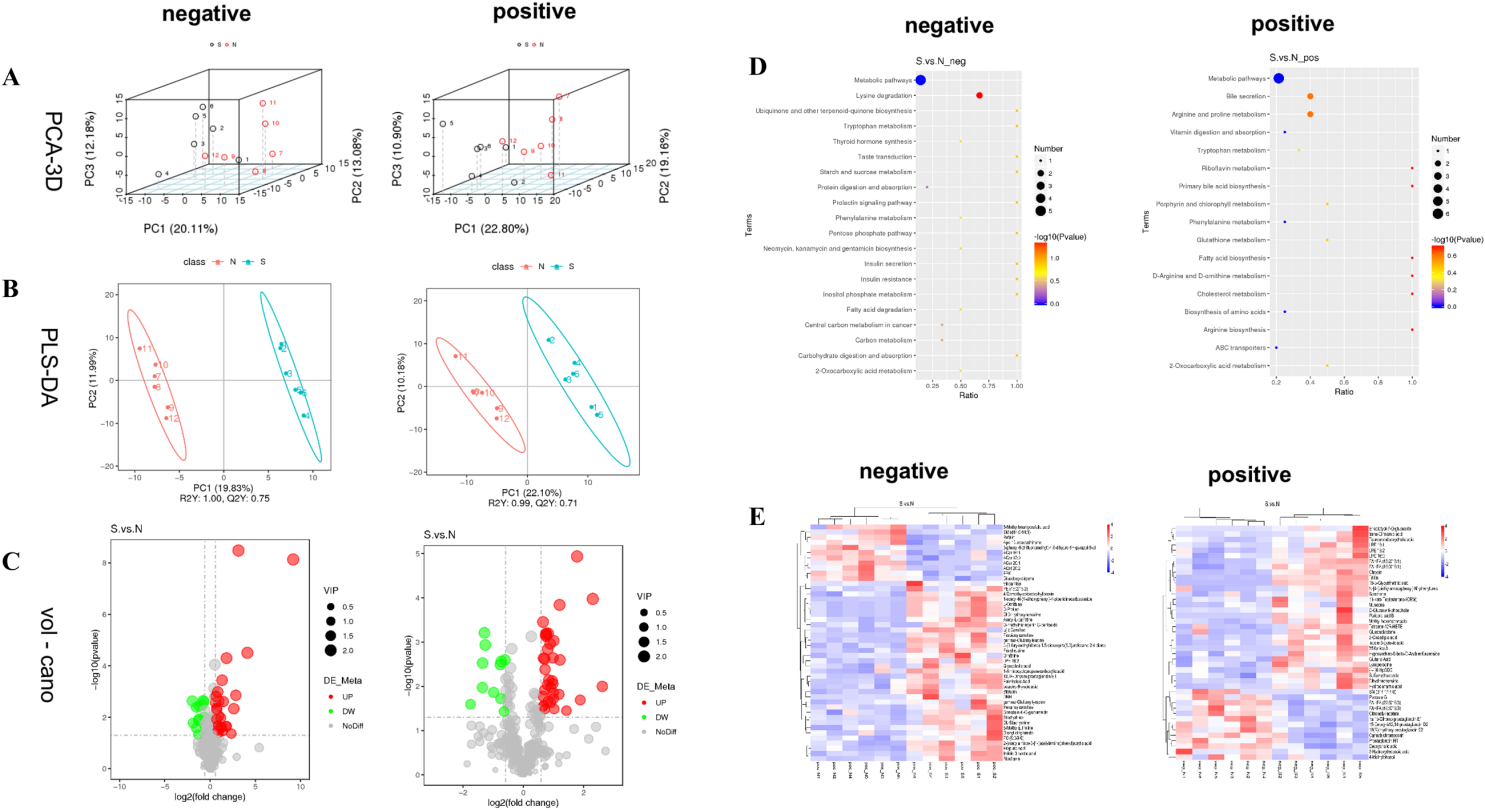
Differential metabolites in plasma were identified in the SNKAF group and the control group. **A** 3D-PCA plot based on data from the control and SNKAF groups obtained in the positive (right)-and negative (left)-ion modes. **B** PLSDA plot based on data from the control and SNKAF groups obtained in the positive (right)-and negative (left)-ion modes. **C** Volcano-plots of metabolites data from the control and SNKAF groups obtained in the positive (right)-and negative (left)-ion modes; **D** VIP plots of fecal samples from the control and SNKAF groups obtained in the positive (right)-and negative (left)-ion modes; **E** Heatmap of difference metabolites data from the control, and SNKAF groups obtained in the positive (right)-and negative (left)-ion modes

To know the metabolic roles of SNKAF in HCC, this paper examined and analyzed differential metabolites in plasma samples from mice. In the basic data analysis, Fig. 5 displays the basal peak intensity (BPI) chromatograms of the serum supernatants of the Control and SNKAF groups, indicating good peak shape and relatively uniform distribution under the test conditions. 3D-PCA analysis (Fig. 6A), PLS-DA (Fig. 6B), Volcano (Fig. 6C) of the control and SNKAF groups were performed with Them EZinfo. Good isolation was realized in both groups, showing improved abnormal metabolism after the administration of SNKAF. Heat map analysis of the total 89 differential metabolites in pos- and neg-ion mode (Figs. 6E), the degree of variation is labeled with various colors, every row stands for a single sample, and every column stands for a metabolite (more information in Additional file 2: Table S2). Following metabolic pathway analysis according the differential metabolites by KEGG (Figs. 6D), five metabolic pathways, biosynthesis of amino acids, phenylalanine metabolism, ABC transporters, and Vitamin digestion and absorption were found(*P-*value = 1). The metabolites included indole-3-acetic acid, bilirubin, l-Ornithine, d-Proline, glycocholic acid, riboflavin, trans-Cinnamic acid, 4-Methylphenol, gluconolactone, d-Glucose 6-phosphate, 2-Oxoadipic acid, Hippuric acid (more information in Additional file 3: Table S3).

### Integrated analysis of metabolomics and network pharmacology

**Fig. 7:**
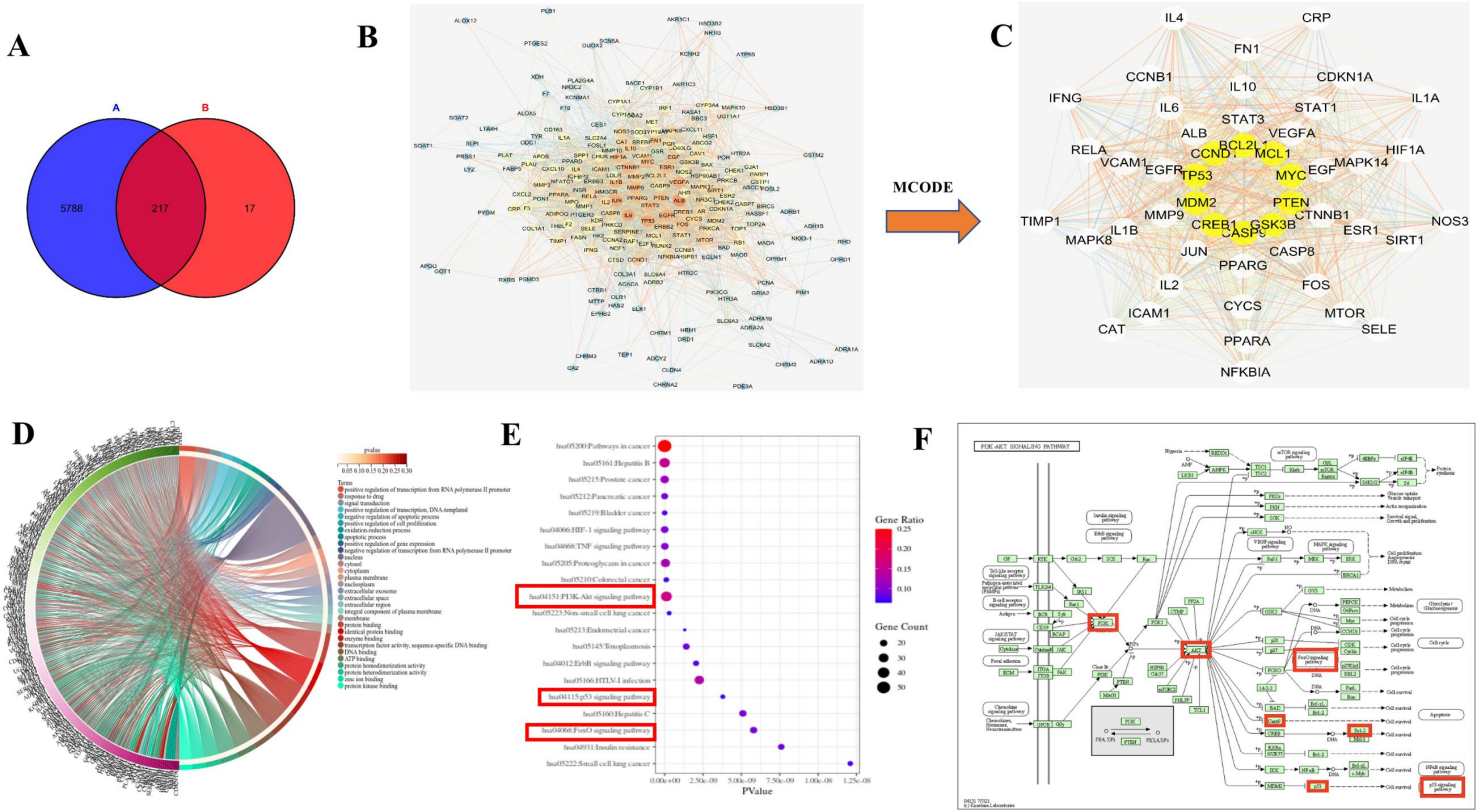
Combined network pharmacology and metabolomics to analyze the target of SNKAF in HCC. **A** The Venn analysis showed the crosstalk of DEGs in two omics, (A: metabolomics, B: network pharmacology); **B** PPI network of HCC targets and **C** Top five clustering graphs from the PPI network of HCC targets. **D** The GO enrichment analysis of potential targets by ClueGO; **E** Top 20 significantly enriched pathways enrichment by KEGG analysis; **F** KEGG pathway suggested that various targets in PI3K/Akt signaling were tightly associated with SNKAF pharmacological action. The red rectangle nodes represent the most significant genes or biological pathways associated with SNKAF pharmacological action

For an integrated vision of the mechanisms of SNKAF against HCC, an interaction network was set up based on metabolomics and network pharmacology. The target genes obtained by differential metabolites intersect with the 234 differential genes obtained cross-network pharmacology, 217 of them overlapped in the Venn diagram analysis (Fig. 7A), The String database online service platform was adopted to perform Protein–protein interaction (PPI) network analysis on the HCC targets, as displayed in Fig. 7B. The yellow nodes in the middle stand for the genes, which may exert significant effects on the genesis and progression of HCC though the MCODE plug in in Cytoscape They included BCL2, MCL1, MYC, PTEN,GSK3B, CASP9, CREB1, MDM2, PT53, CCND1 (Fig. 3C). The top 10 important terms in GO functional enrichment and the top 20 important KEGG pathways are displayed in Fig. 7D and E, respectively. KEGG analysis (*P* < 0.05) suggested that various cancer-associated pathways were largely involved in the mechanisms of SNKAF, such as apoptosis, cell cycle, P53, HIF-1, ErbB, FoxO, PI3K/Akt signaling pathways. Remarkably, different targets in PI3K/Akt signaling can be boosted by a number of stress signals, such as DNA harm caused by the p53 signaling. Consequently, results in cell cycle arrest and apoptosis [38], the cell cycle effect of the FoxO signaling pathway [39], and even influencing apoptotic markers such as Bax, Bcl-2.

### Validation the SNKAF as a potential anti-cancer inhibitor through the PI3K/Akt/P53/FoxO axis in HCCs

**Fig. 8:**
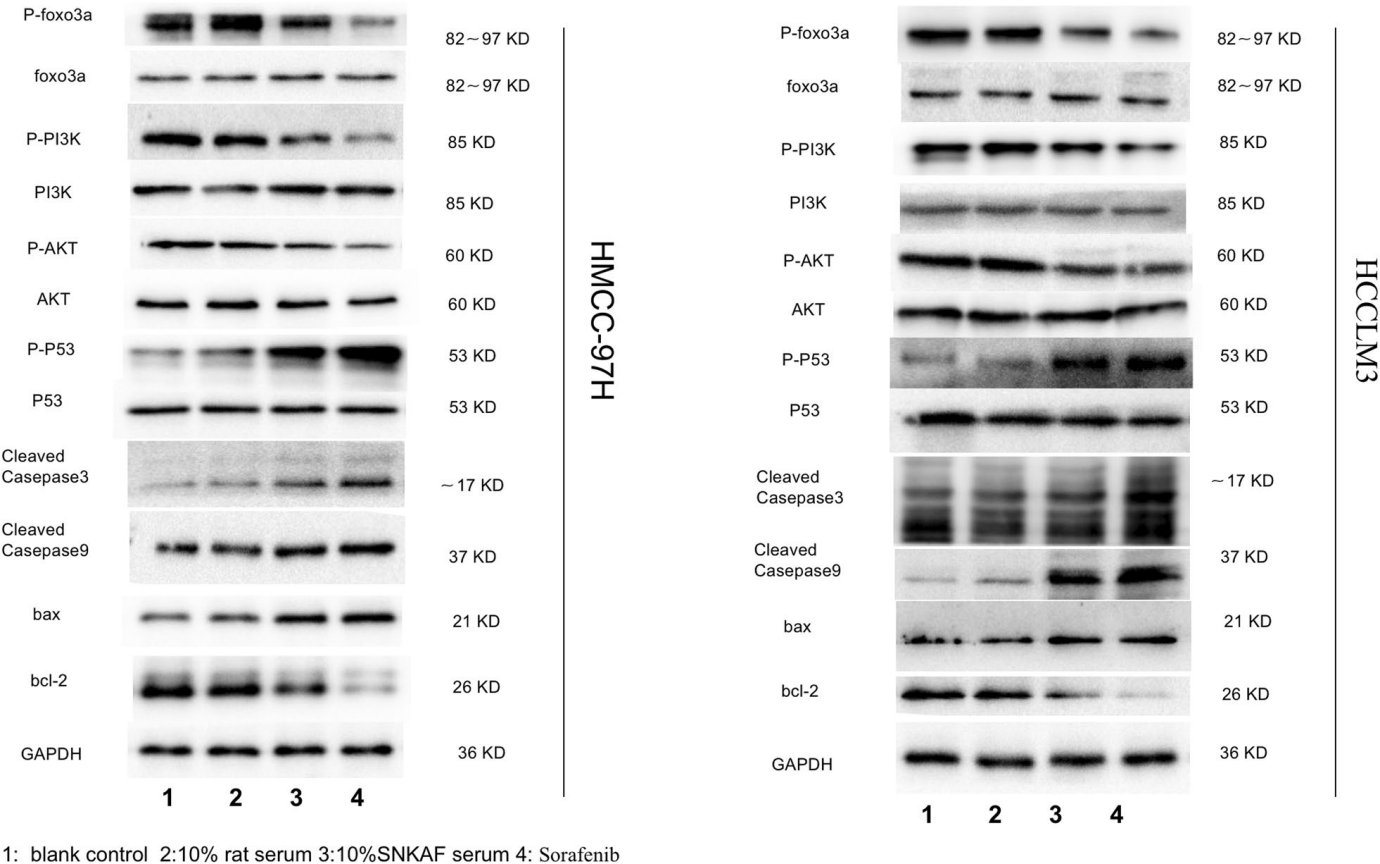
Validation and identification of SNKAF as a anti-cancer inhibitor in suppressing HCC apoptosis and proliferation through the PI3K/Akt/P53/FoxO Axis. Protein expression by Western blot assay. The expression of p-Fox3a/Fox3a, p-PI3K/PI3K, p-Akt/Akt, p-P53/P53, Bax, Bcl-2,cleaved-caspase-9 and cleaved-caspase-3 using western blotting analysis after indicated treatment in HCCLM3 and MHCC97H cells; Lanes 1–4: blank control; 10% rat serum; 10% SNKAF serum; sorafenib

**Fig. 9:**
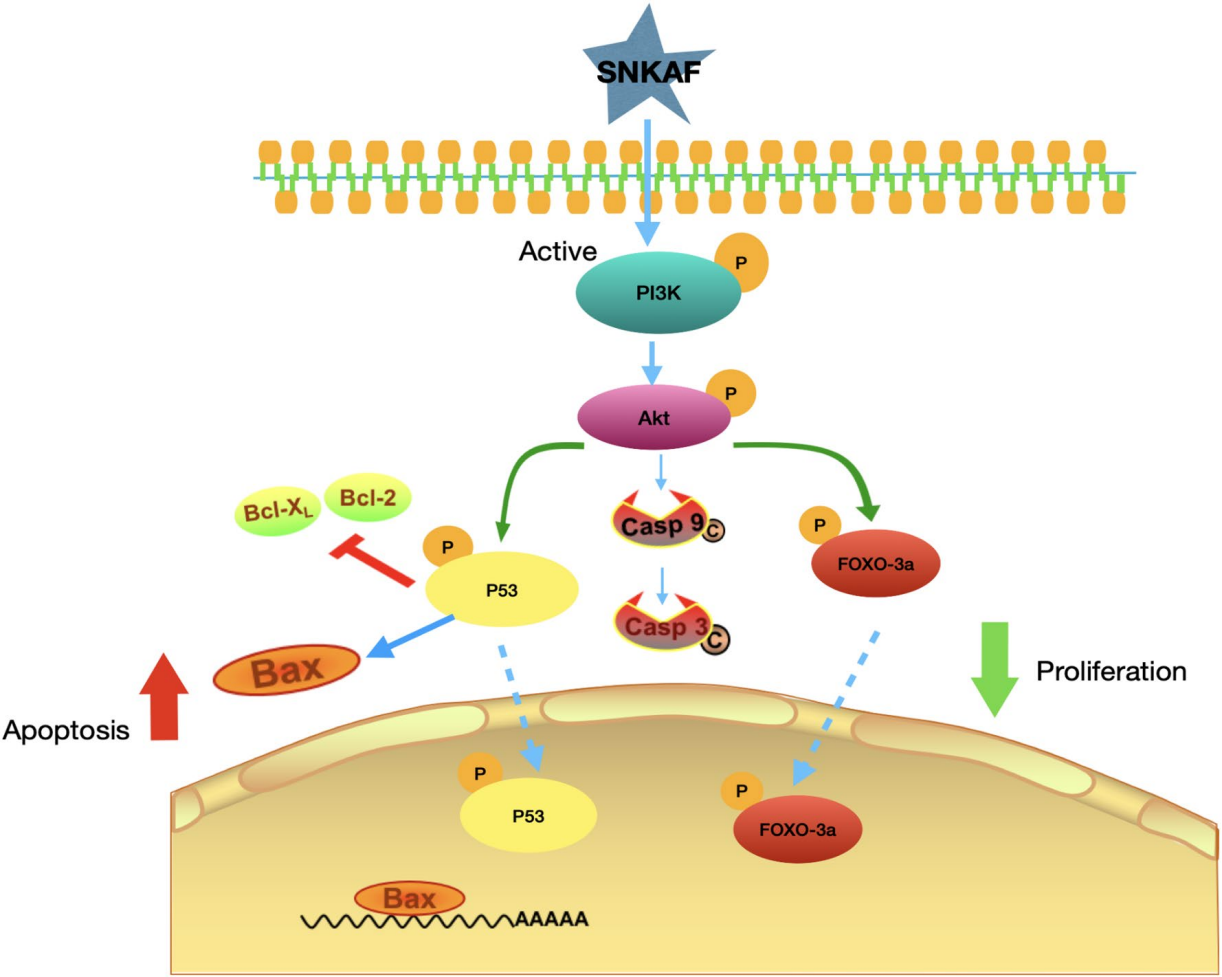
The proposed mechanisms of SNKAF-inhibited proliferation and induced apoptosis

Metabolomics and KEGG analysis indicated that apoptosis was necessary for the anti-cancer function of SNKAF; hence, the role of SNKAF in apoptosis flux in HCCs was assessed. As shown in Fig. 7E, the panel displays the PI3K/Akt signaling pathway from KEGG result. The red rectangle nodes stand for the most prominent genes associated with SNKAF pharmacological actions. Western blot analysis (Fig. 8) showed that, of the various treatment groups, injection of sorafenib or SNFAF serum, the 10% rat serum had no obvious effect on the protein expression of the ratios of p-Fox3a/Fox3a, p-PI3K/PI3K, p-Akt/Akt, and p-P53/P53 and cleaved-caspase-9, cleaved-caspase-3, Bax, Bcl-2 on HMCC-97H. However, 10% SNFAF serum and sorafenib promoted the protein expression of cleaved-caspase-9, cleaved-caspase-3, Bax and the ratios of p-P53/P53. In contrast, 10% SNFAF serum and sorafenib diminished the protein expression of Bcl-2 and the ratios of p-Fox3a/Fox3a, p-PI3K/PI3K, and p-Akt/Akt. In addition, a similar trend was observed in HCCLM3 cells. These results indicate that the anti-cancer effects of SNKAF decoction on HCC were associated with the cell proliferation and apoptotic involved PI3K/Akt pathway axis with the downstream cell survival P53 signal pathway, cell cycle progression, and FoxO signal pathway (Fig. 9).

## Discussion

Currently, the world has extensively focused on TCM for the treatment or prevention different diseases [40], such as cancers [41]. SNKAF decoction exerts a significant effect by alleviating the symptoms of liver patients in the clinic. However, to date, there is no elucidation of its potential mechanism, particularly in terms of its active compound and the core target. Therefore, network pharmacology and metabolomics were combined with biological approaches to understand the potential mechanism of SNKAF decoction.

SNKAF formula contains 15 herbal compounds, and there are more than 197 active compounds based on pharmacological research (Additional file 1: Table S1). The verification of whether these compounds have a pharmacodynamic effect on the body is quite complex. Serum pharmacology eliminates the interference of the physical and chemical properties of TCM preparations and shows the last process of digesting and absorbing TCM in the gastrointestinal tract. There was a study on the administration of Acanthopanax senticosus to mice, and 12 prototype and 9 metabolic components were found, which were screened faster than 131 components in vitro [42]. Moreover, this method reflects the real effective function of drug components affecting the body. We used the SNKAF serum as the research object to study the pharmacodynamic mechanism. There were 89 different metabolites produced after the administration of SNKAF, of which 24 metabolic were downregulated, and 65 were upregulated.

Biosynthesis of amino acids plays an important role in many research activities in cancer, such as Pro, which regulates cytoplasmic balance and is a significant component of collage. Its biosynthetic is necessary for remodeling the tumor microenvironment and extracellular matrix to boost the reprogramming and proliferation of cancer cells. Besides, Pro can produce ATP for cell growth through the TCA cycle during catabolism. There are research on at the primary growth of HCC. Amino acids, originating from the degradation of proteins in liver tissue or other tissues, may increase greatly and are then taken up by amino acid transporters in liver cancer tissue to aid rapid proliferation [43]. Phenylalanine is transformed into tyrosine by the catalytic oxidation of phenylalanine hydroxylase. Tyrosine takes part in glucose and fat metabolism in the body, which are the main energy sources utilized by cancer cells ro grow rapidly [44]. Phenylalanine level were significant increase in hepatocarcinoma with lung metastasis, which may be related with decreased catabolism in the liver [45]. Therefore, in our study, we evaluated the significant difference in of amino acids biosynthesis and phenylalanine metabolism of the serum metabolites in SNKAF decoction. It indicated that amino acids biosynthesis and phenylalanine metabolism are the main metabolic pathways taking part in various cellular processes, including cancer cell proliferation, migrate and so on.

As multi-target measures for cancer treatment attract increasing attention, research strategies are relying on network pharmacology combine with metabolomics to study the intricate associations between TCM formulae and the related targets and diseases. This is further combined with bioinformatic analysis to explore the disease mechanisms and intervention strategies [45, 46] by constructing the PPI network for the common targets of metabolomics with network pharmacology. These top 10 genes (Fig. 3C) are mainly related to cell proliferation, apoptosis, oxidation–reduction process, regulation of transcription from RNA polymerase II promoter, signal transduction via proteoglycans in cancer, hypoxia-inducible factor 1 (HIF-1), TNF, ErbB, PI3K/Akt, P53, and FoxO signaling pathways. The antioxidant effect was mainly reported in the HIF-1 signaling pathway. It has been reported that cell apoptosis was promoted when the HIF-1 signaling pathway was activated in epidermal HaCaT cells under an oxidative-stress microenvironment [47]. As a proinflammatory cytokine, TNF increases the key molecules driving oncogenic events by activating different cells, so as to launch various malignant processes [36]. Furthermore, intracellular signaling pathways and extracellular growth factor ligands are bound through the ERBB family, which is a member of receptor tyrosine kinases, to regulate various biological reactions and can activate the PI3K pathway [48].

As cell cycle and cell death signaling pathways exert significant effects on cancer growth, they act as potential cancer therapeutic targets [49]. The in-vivo assay revealed that SNKAF could effectively suppress the growth and prolong the survival of subcutaneous tumors in mice (Fig. 1). The in-vitro research was conducted to verify the anti-cancer effect on cells (Fig. 3), including inhibition of HCC growth, progression (Figs. 2 and 3), migration, and invasion (Fig. 2). Similar to the previous bioinformatic assay, 5 core targets (BCL2, GSK3B, CASP9, MDM2, and PT53) were closely related to three signaling pathways (PI3K/Akt, P53, and FoxO signaling pathways). The mechanisms are closely associated with the pathological cell cycle and death changes of HCC. The PI3K/Akt pathway exerts a significant effect on cell survival, proliferation, migration, metabolism, and apoptosis among different tumor kinds, including HCC [35, 50]. The compounds of SNKAF were boosted upon the binding of the signaling molecules and consequently provoked the related receptors at the surface of the cell membrane. This resulted in the conformational variations at the PI3K receptor and the follow-up recruitment phosphorylation. Following activation, PI3k induced the activation of a core signaling kinase Akt, which controls some downstream effector molecules like glycogen synthase kinase-3β (GSK3β), driving protein and lipid synthesis, and cell development [51, 52]. It also induced the activation of the forkhead box O (FOXO) family, which participates in various cellular physiological processes and regulates cell proliferation, cell cycle [53, 54]. Besides, the activated Akt may inhibit apoptosis and drives cell survival via the follow-up modulation of different target molecules, including the Bcl-2 family of proteins [49, 55, 56]. The activation of the Akt can promote the up-regulation of MDM2, a main negative regulators of P53, that may strengthen the degradation of P53 and inhibit its phosphorylation to prevent apoptosis and modulate the cell cycle [57].

## Conclusion

This study's findings indicated that SNKAF efficacy was via the synergistic role of multi-compounds, multi-targets, and multi-pathways. To verify the molecular mechanism of effect of SNKAF on HCC, western blotting assays were utilized. The results showed that SNKAF reduced the levels of phosphorylated PI3K, inhibited cell proliferation and recruitment in the cytoplasm, and further influenced phosphorylated Akt. On the one hand, SNKAF decreased the levels of p-PI3K and p-Akt, which in turn targets downstream regulatory markers of apoptosis, like caspase 9, Bax, Bim, Bcl-XL, leading the occurrence of apoptosis in HCC cells. On the other hand, SNKAF regulated the progress of apoptosis and proliferation through mediates the interaction of P53 and Foxo. Therefore, our study is novel in that it is the first to explore the association between the anti-apoptotic signaling pathway axis in HCC following SNKAF decoction.

## Supplementary Information

**Additional file 1: Table S1.** The potential active compounds from SNKAF Prescription.

**Additional file 2: Table S2.** 89 differentially metabolized compounds.

**Additional file 3: Table S3.** Metabolic pathways and corresponding metabolites.

## Data Availability

The datasets used and/or analysed during the current study are available from the corresponding author on reasonable request.

## Abbreviations

SNKAF: Si Ni Kang Ai Fang
DAVID: Database for Annotation, Visualization and Integrated Discovery
GO: Gene Ontology
H&E staining: Hematoxylin–eosin staining
IC50: The half maximal inhibitory concentration
IHC: Immunohistochemistry
KEGG: Kyoto Encyclopedia of Genes and Genomes
PI: Propidium iodide
PPI: Protein–protein interaction
TCM: Traditional Chinese medicine
TCMID: Traditional Chinese Medicine Integrated Database
TCMSP: Traditional Chinese Medicine Systems Pharmacology
TUNEL: Terminal deoxynucleotidyl transferase(TdT)-mediated dUTP nick end labeling
ESI-MS^n^: Electrospray ionization–mass spectrometry
