# Supplementary material for: Metabolomics and integrated network pharmacology analysis reveal SNKAF decoction suppresses cell proliferation and induced cell apoptisis in hepatocellular carcinoma via PI3K/Akt/P53/FoxO signaling axis
Source: Chin Med. 2022 Jun 20;17:76. doi: 10.1186/s13020-022-00628-1 (PMC9208213; doi:10.1186/s13020-022-00628-1)
Supplement: Supplementary file 1 — Additional file 1: Table S1. The potential active compounds from SNKAF Prescription. [file 13020_2022_628_MOESM1_ESM.docx]

**Supplement Table 1 the potential active compounds from SNKAF Prescription**

| **Number** | | | **Compounds** | | **OB (%)** | | **DL** | | **Source** | | **Structure** | |  |
| --- | --- | --- | --- | --- | --- | --- | --- | --- | --- | --- | --- | --- | --- |
| MOL001645 | | | Linoleyl acetate | | 42.1 | | 0.2 | | CH | | 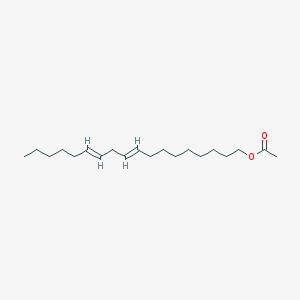   \|  \| \| --- \| | | |
| MOL004644 | | | Sainfuran | | 79.91 | | 0.23 | | CH | | 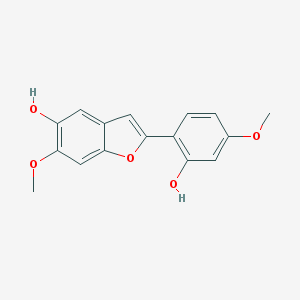   \|  \| \| --- \| | | |
| MOL000422 | | | kaempferol | | 41.88 | | 0.24 | | CH， BS，GC | | 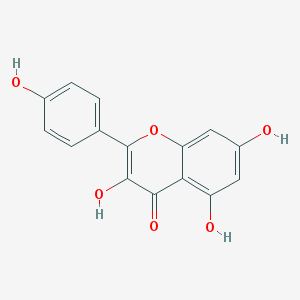   \|  \| \| --- \| | | |
| MOL000098 | | | quercetin | | 46.43 | | 0.28 | | CH，GC，BZL，LK，BHSSC | | 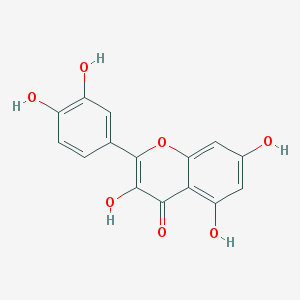   \|  \| \| --- \| | | |
| MOL004628 | | | Octalupine | | 47.82 | | 0.28 | | CH | | 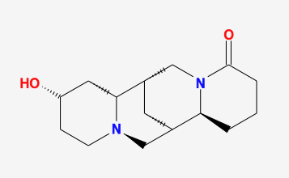   \|  \| \| --- \| | | |
| MOL004648 | | | Troxerutin | | 31.6 | | 0.28 | | CH | | 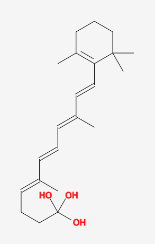   \|  \| \| --- \| | | |
| MOL000354 | | | isorhamnetin | | 49.6 | | 0.31 | | CH，GC | | 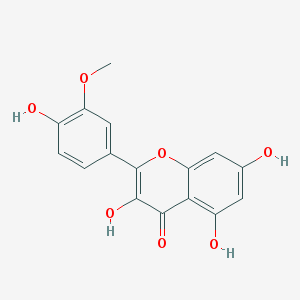   \|  \| \| --- \| | |  |
| MOL000490 | | | petunidin | | 30.05 | | 0.31 | | CH | | 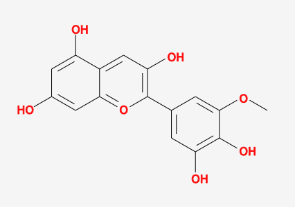 | |  |
| MOL004609 | | | Areapillin | | 48.96 | | 0.41 | | CH | | 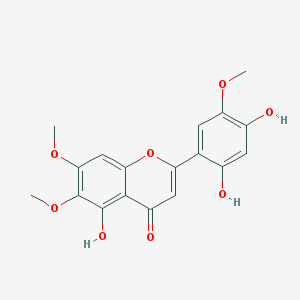 | |  |
| MOL004624 | | | Longikaurin A | | 47.72 | | 0.53 | | CH | | \| 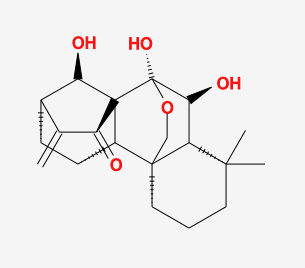 \| \| --- \| | |  |
| MOL004598 | | | 3,5,6,7-tetramethoxy-2-(3,4,5-trimethoxyphenyl)chromone | | 31.97 | | 0.59 | | CH | | 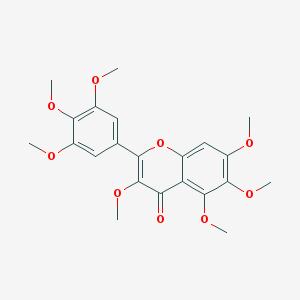 | |  |
| MOL004702 | | | saikosaponin c_qt | | 30.5 | | 0.63 | | CH | | 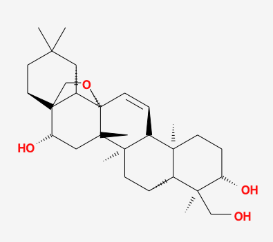 | |  |
| MOL013187 | | | Cubebin | | 57.13 | | 0.64 | | CH | | 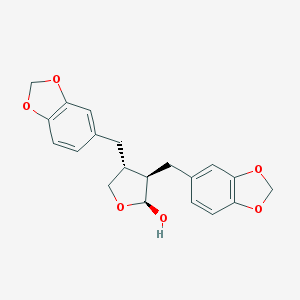   \|  \| \| --- \| | |  |
| MOL004653 | | | (+)-Anomalin | | 46.06 | | 0.66 | | CH | | 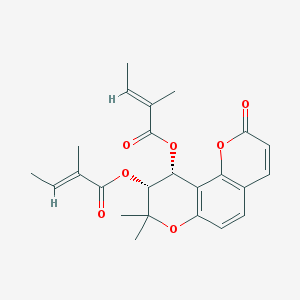 | |  |
| MOL000449 | | | Stigmasterol | | 43.83 | | 0.76 | | CH,DS，YYR，BHSSC，BZL，SCG | | 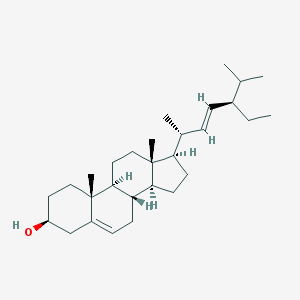 | |  |
| MOL004718 | | | α-spinasterol | | 42.98 | | 0.76 | | CH | | 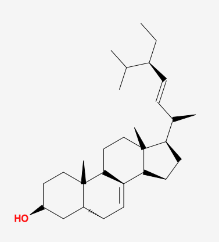 | |  |
| MOL001910 | | 11alpha,12alpha-epoxy-3beta-23-dihydroxy-30-norolean-20-en-28,12beta-olide | | | 64.77 | | 0.38 | | BS | | 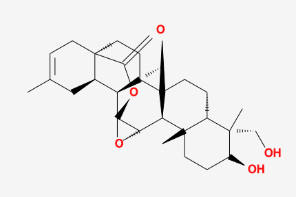 | |  |
| MOL001919 | | (3S,5R,8R,9R,10S,14S)-3,17-dihydroxy-4,4,8,10,14-pentamethyl-2,3,5,6,7,9-hexahydro-1H-cyclopenta[a]phenanthrene-15,16-dione | | | 43.56 | | 0.53 | | BS | | 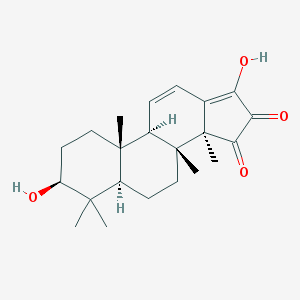 | |  |
| MOL000359 | | sitosterol | | | 36.91 | | 0.75 | | BS，GC，YYR，BZL，LK | | 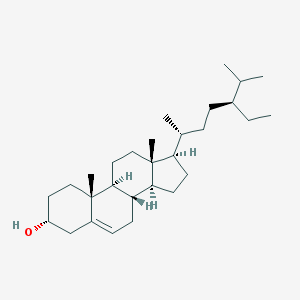 | |  |
| MOL000358 | | beta-sitosterol | | | 36.91 | | 0.75 | | BS，SCG，BZL，TR，BHSSC | | 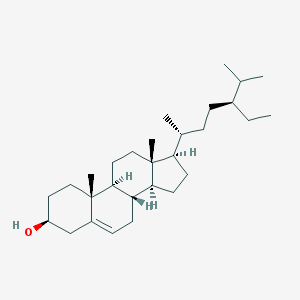 | |  |
| MOL000211 | | | | Mairin | | 55.38 | | 0.78 | | BS，GC | | 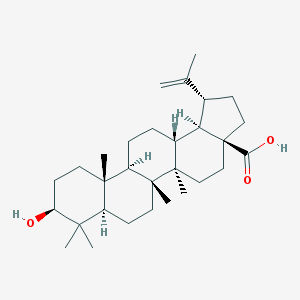 | |
| MOL004328 | | | | naringenin | | 59.29 | | 0.21 | | ZS，GC | | 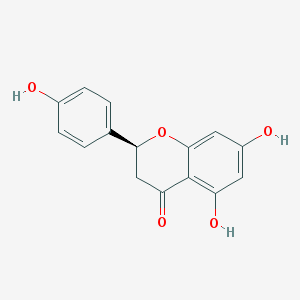 | |
| MOL001941 | | | | Ammidin | | 34.55 | | 0.22 | | ZS | | 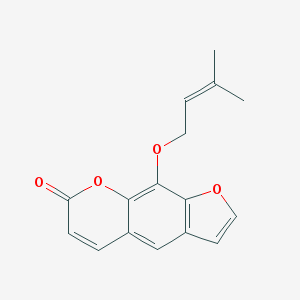 | |
| MOL005849 | | | | didymin | | 38.55 | | 0.24 | | ZS | | 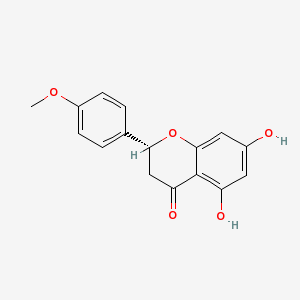 | |
| MOL002914 | | | | Eriodyctiol (flavanone) | | 41.35 | | 0.24 | | ZS | | 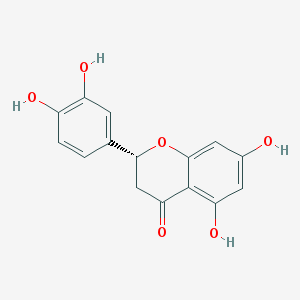   \|  \| \| --- \| | |
| MOL000006 | | | | luteolin | | 36.16 | | 0.25 | | ZS，DS，BZL | | 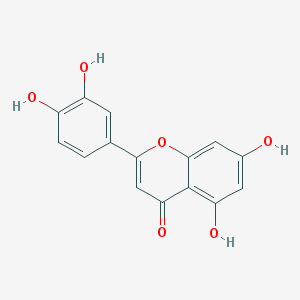 | |
| MOL001798 | | | | neohesperidin_qt | | 71.17 | | 0.27 | | ZS | | 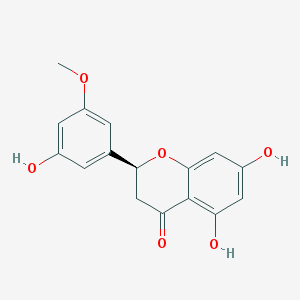 | |
| MOL005100 | | | | 5,7-dihydroxy-2-(3-hydroxy-4-methoxyphenyl)chroman-4-one | | 47.74 | | 0.27 | | ZS | | 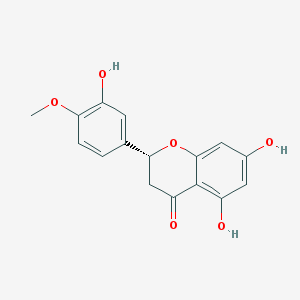 | |
| MOL013433 | | | | prangenin hydrate | | 72.63 | | 0.29 | | ZS | | 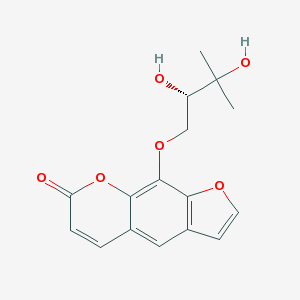 | |
| MOL013430 | | | | Prangenin | | 43.6 | | 0.29 | | ZS | | 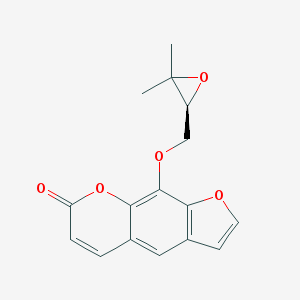   \|  \| \| --- \| | |
| MOL013279 | | | | 5,7,4'-Trimethylapigenin | | 39.83 | | 0.3 | | ZS | | 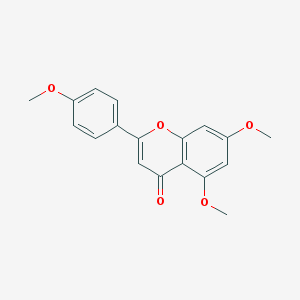 | |
| MOL013437 | | | | 6-Methoxy aurapten | | 31.24 | | 0.3 | | ZS | | 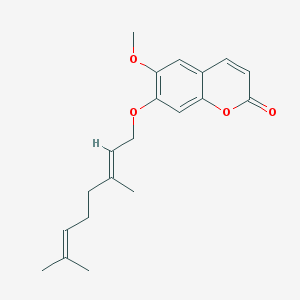 | |
| MOL013436 | | | | isoponcimarin | | 63.28 | | 0.31 | | ZS | | 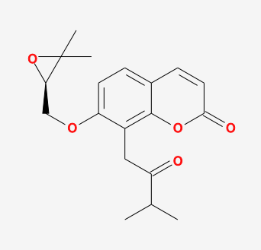 | |
| MOL013435 | | | | poncimarin | | 63.62 | | 0.35 | | ZS | | 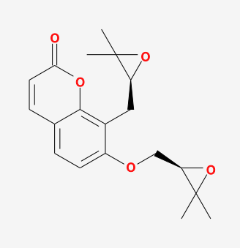 | |
| MOL007879 | | | | Tetramethoxyluteolin | | 43.68 | | 0.37 | | ZS | | \| 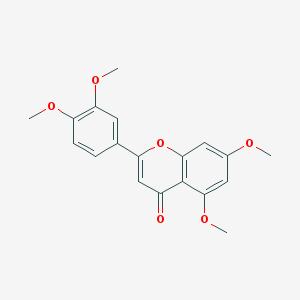 \| \| --- \| | |
| MOL009053 | | | | 4-[(2S,3R)-5-[(E)-3-hydroxyprop-1-enyl]-7-methoxy-3-methylol-2,3-dihydrobenzofuran-2-yl]-2-methoxy-phenol | | 50.76 | | 0.39 | | ZS | | 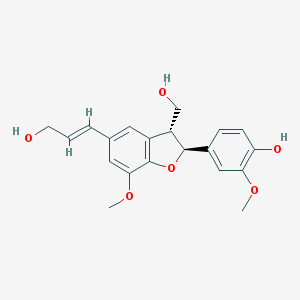 | |
| MOL013277 | | | | [Isosinensetin](http://tcmspw.com/molecule.php?qn=13277) | | 51.15 | | 0.44 | | ZS | | 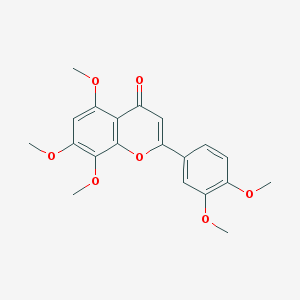 | |
| MOL001803 | | | | [Sinensetin](http://tcmspw.com/molecule.php?qn=1803) | | 50.56 | | 0.45 | | ZS | | 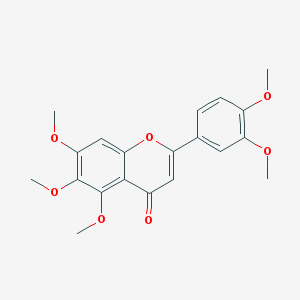 | |
| MOL005828 | | | | [nobiletin](http://tcmspw.com/molecule.php?qn=5828) | | 61.67 | | 0.52 | | ZS | | 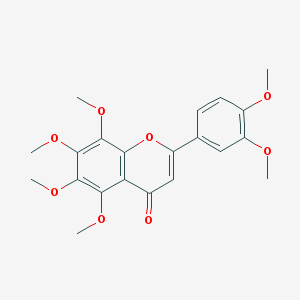 | |
| MOL013352 | | | | [Obacunone](http://tcmspw.com/molecule.php?qn=13352) | | 43.29 | | 0.77 | | ZS | | 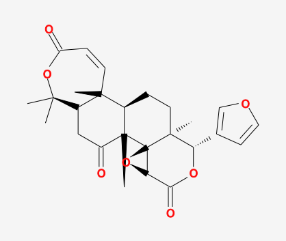 | |
| MOL004941 | | | | (2R)-7-hydroxy-2-(4-hydroxyphenyl)chroman-4-one | | 71.12 | | 0.18 | | GC | | 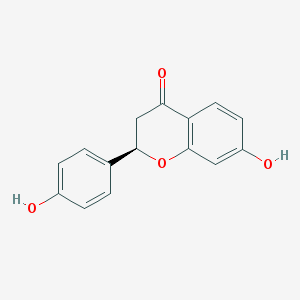 | |
| MOL001792 | | | | DFV | | 32.76 | | 0.18 | | GC | | \| 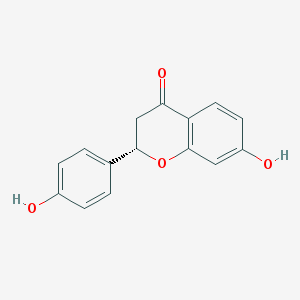 \| \| --- \| | |
| MOL004835 | | | | Glypallichalcone | | 61.6 | | 0.19 | | GC | | 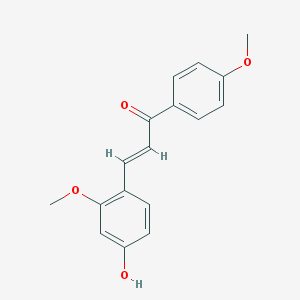 | |
| MOL004841 | | | | Licochalcone B | | 76.76 | | 0.19 | | GC | | 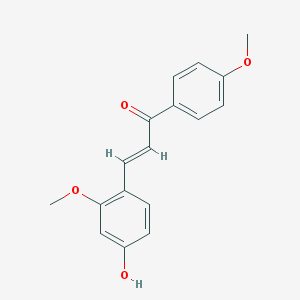 | |
| MOL004985 | | | | icos-5-enoic acid | | 30.7 | | 0.2 | | GC | | 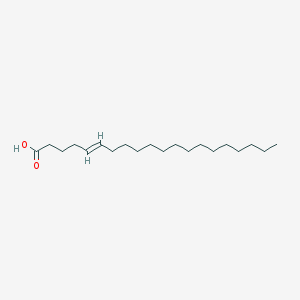 | |
| MOL004996 | | | | gadelaidic acid | | 30.7 | | 0.2 | | GC | | 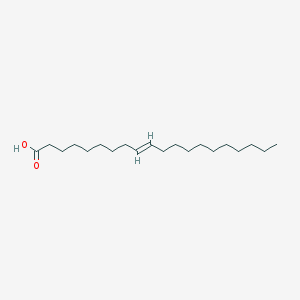 | |
| MOL003896 | | | | 7-Methoxy-2-methyl isoflavone | | 42.56 | | 0.2 | | GC，DS | | 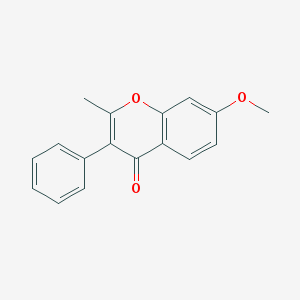 | |
| MOL000500 | | | | Vestitol | | 74.66 | | 0.21 | | GC | | 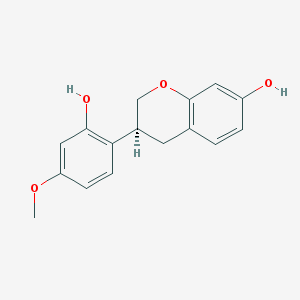 | |
| MOL004957 | | | | HMO | | 38.37 | | 0.21 | | GC | | 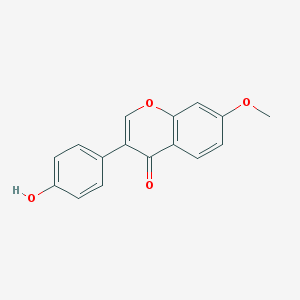 | |
| MOL000392 | | | | formononetin | | 69.67 | | 0.21 | | GC | | \| 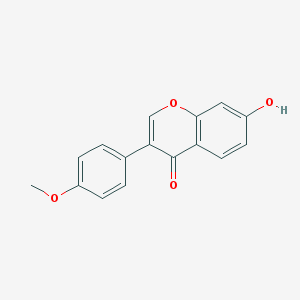 \| \| --- \| | |
| MOL000417 | | | | Calycosin | | 47.75 | | 0.24 | | GC | | 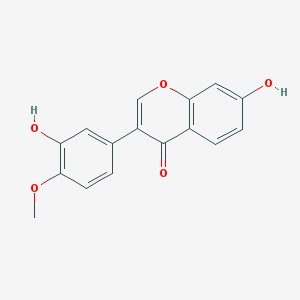 | |
| MOL004991 | | | | 7-Acetoxy-2-methylisoflavone | | 38.92 | | 0.26 | | GC | | 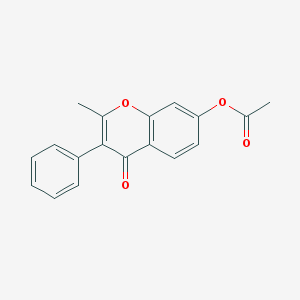 | |
| MOL004990 | | | | 7,2',4'-trihydroxy－5-methoxy-3－arylcoumarin | | 83.71 | | 0.27 | | GC | | 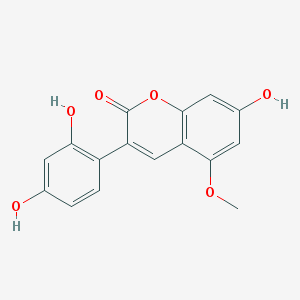 | |
| MOL000497 | | | | licochalcone a | | 40.79 | | 0.29 | | GC | | 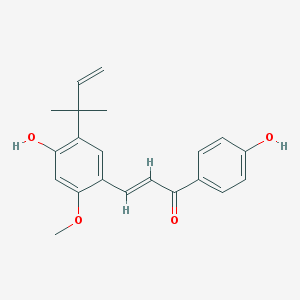 | |
| MOL000239 | | | | Jaranol | | 50.83 | | 0.29 | | GC | | 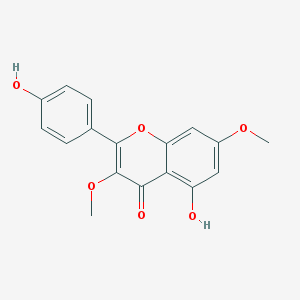 | |
| MOL005016 | | | | Odoratin | | 49.95 | | 0.3 | | GC | | 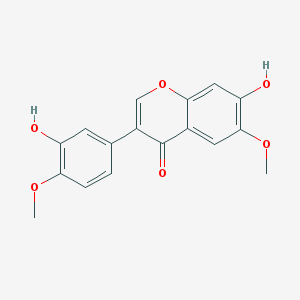 | |
| MOL004898 | | | | (E)-3-[3,4-dihydroxy-5-(3-methylbut-2-enyl)phenyl]-1-(2,4-dihydroxyphenyl)prop-2-en-1-one | | 46.27 | | 0.31 | | GC | | 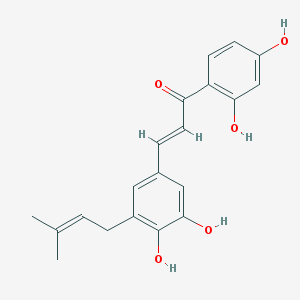 | |
| MOL004910 | | | | Glabranin | | 52.9 | | 0.31 | | GC | | 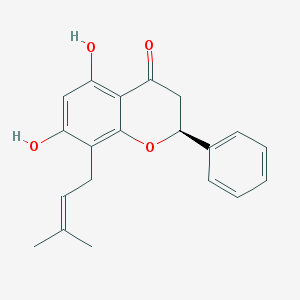 | |
| MOL004945 | | | | (2S)-7-hydroxy-2-(4-hydroxyphenyl)-8-(3-methylbut-2-enyl)chroman-4-one | | 36.57 | | 0.32 | | GC | | 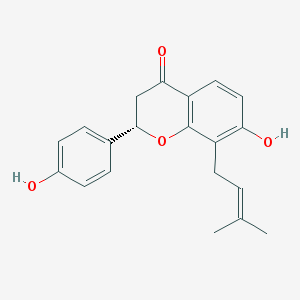 | |
| MOL004848 | | | | licochalcone G | | 49.25 | | 0.32 | | GC | | \| 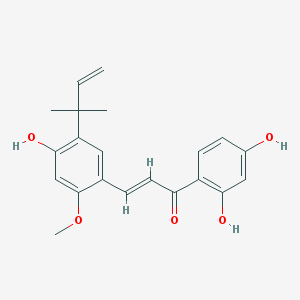 \| \| --- \| | |
| MOL004980 | | | | Inflacoumarin A | | 39.71 | | 0.33 | | GC | | 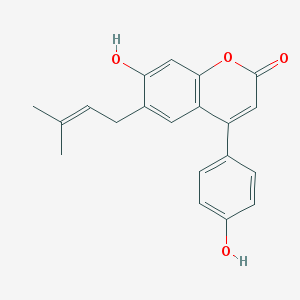 | |
| MOL004961 | | | | Quercetin der. | | 46.45 | | 0.33 | | GC | | 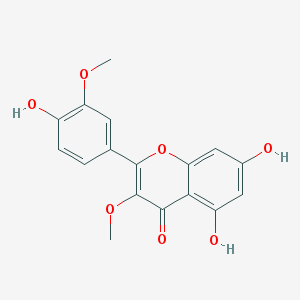 | |
| MOL002565 | | | | Medicarpin | | 49.22 | | 0.34 | | GC | | 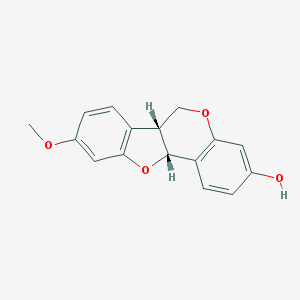 | |
| MOL004829 | | | | Glepidotin B | | 64.46 | | 0.34 | | GC | | 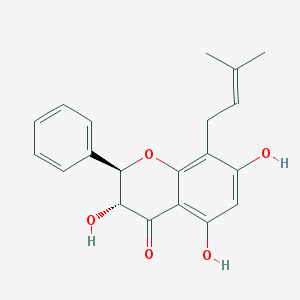 | |
| MOL004828 | | | | Glepidotin A | | 44.72 | | 0.35 | | GC | | \| 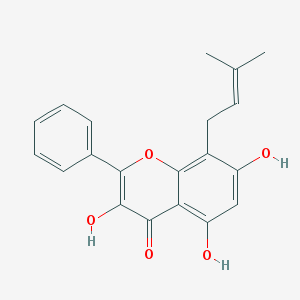 \| \| --- \| | |
| MOL004815 | | | | (E)-1-(2,4-dihydroxyphenyl)-3-(2,2-dimethylchromen-6-yl)prop-2-en-1-one | | 39.62 | | 0.35 | | GC | | 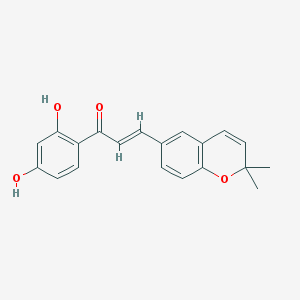 | |
| MOL004907 | | | | Glyzaglabrin | | 61.07 | | 0.35 | | GC | | 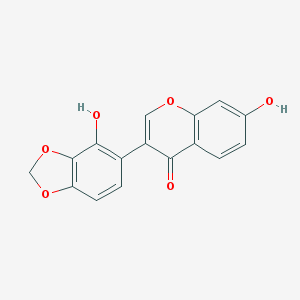 | |
| MOL004882 | | | | Licocoumarone | | 33.21 | | 0.36 | | GC | | 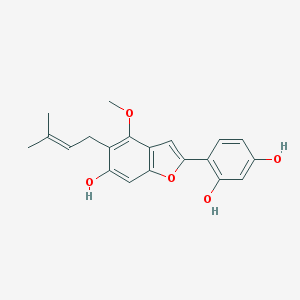 | |
| MOL003656 | | | | Lupiwighteone | | 51.64 | | 0.37 | | GC | | 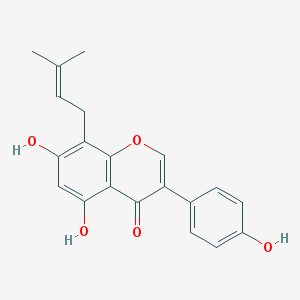 | |
| MOL005020 | | | | dehydroglyasperins C | | 53.82 | | 0.37 | | GC | | \| 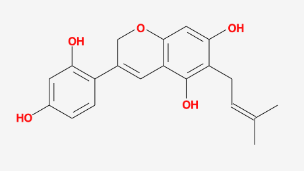 \| \| --- \| | |
| MOL004915 | | | | Eurycarpin A | | 43.28 | | 0.37 | | GC | | 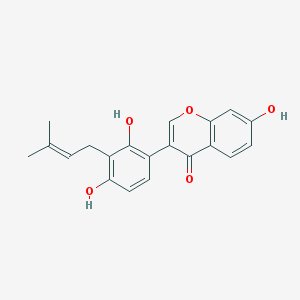 | |
| MOL004838 | | | | 8-(6-hydroxy-2-benzofuranyl)-2,2-dimethyl-5-chromenol | | 58.44 | | 0.38 | | GC | | 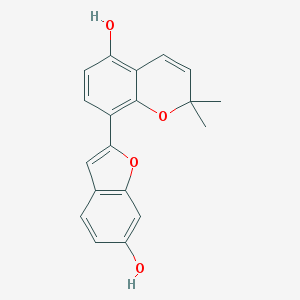 | |
| MOL005000 | | | | Gancaonin G | | 60.44 | | 0.39 | | GC | | 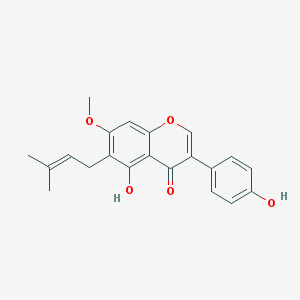 | |
| MOL004811 | | | | Glyasperin C | | 45.56 | | 0.4 | | GC | | 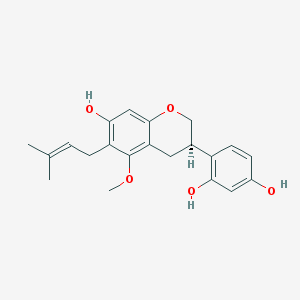 | |
| MOL004856 | | | | Gancaonin A | | 51.08 | | 0.4 | | GC | | 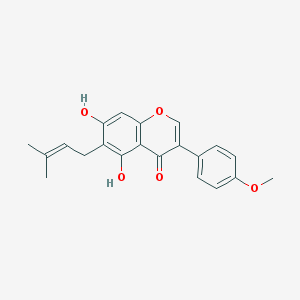 | |
| MOL004993 | | | | 8-prenylated eriodictyol | | 53.79 | | 0.4 | | GC | | 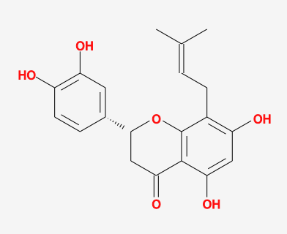 | |
| MOL004864 | | | | 5,7-dihydroxy-3-(4-methoxyphenyl)-8-(3-methylbut-2-enyl)chromone | | 30.49 | | 0.41 | | GC | | 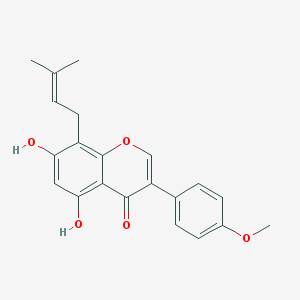 | |
| MOL004989 | | | | 6-prenylated eriodictyol | | 39.22 | | 0.41 | | GC | | 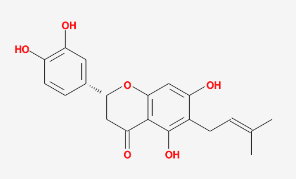 | |
| MOL004863 | | | | 3-(3,4-dihydroxyphenyl)-5,7-dihydroxy-8-(3-methylbut-2-enyl)chromone | | 66.37 | | 0.41 | | GC | | 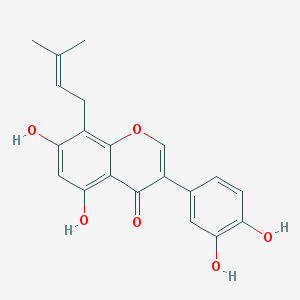 | |
| MOL004935 | | | | Sigmoidin-B | | 34.88 | | 0.41 | | GC | | 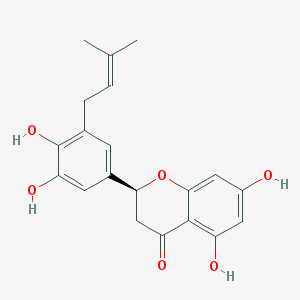 | |
| MOL004866 | | | | 2-(3,4-dihydroxyphenyl)-5,7-dihydroxy-6-(3-methylbut-2-enyl)chromone | | 44.15 | | 0.41 | | GC | | 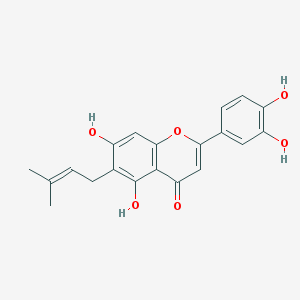 | |
| MOL004883 | | | | Licoisoflavone | | 41.61 | | 0.42 | | GC | | 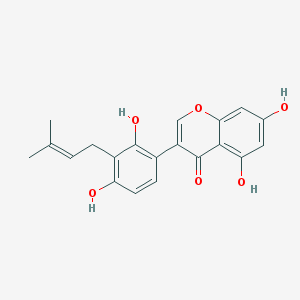 | |
| MOL004949 | | | | Isolicoflavonol | | 45.17 | | 0.42 | | GC | | 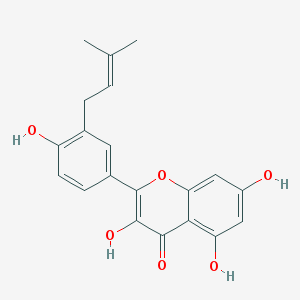 | |
| MOL004814 | | | | Isotrifoliol | | 31.94 | | 0.42 | | GC | | 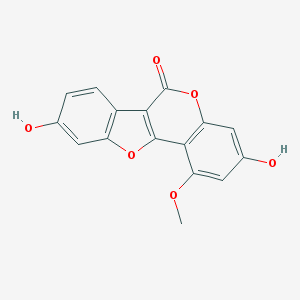 | |
| MOL004913 | | | | 1,3-dihydroxy-9-methoxy-6-benzofurano[3,2-c]chromenone | | 48.14 | | 0.43 | | GC | | 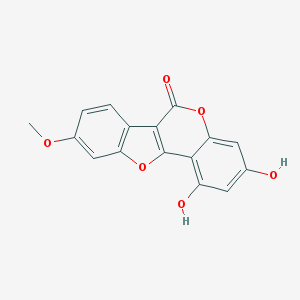 | |
| MOL004849 | | | | 3-(2,4-dihydroxyphenyl)-8-(1,1-dimethylprop-2-enyl)-7-hydroxy-5-methoxy-coumarin | | 59.62 | | 0.43 | | GC | | 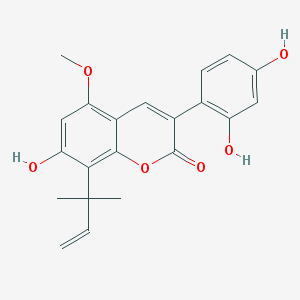 | |
| MOL004808 | | | | glyasperin B | | 65.22 | | 0.44 | | GC | | 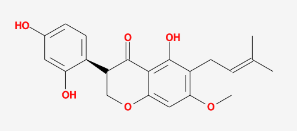 | |
| MOL004911 | | | | Glabrene | | 46.27 | | 0.44 | | GC | | 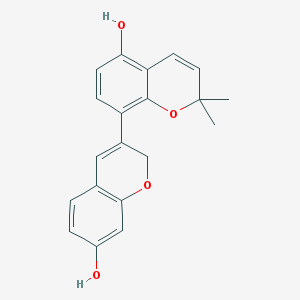 | |
| MOL004833 | | | | Phaseolinisoflavan | | 32.01 | | 0.45 | | GC | | 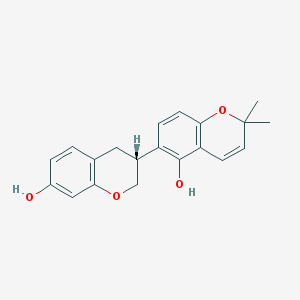 | |
| MOL004857 | | | | Gancaonin B | | 48.79 | | 0.45 | | GC | | 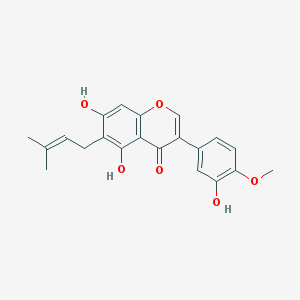 | |
| MOL004908 | | | | Glabridin | | 53.25 | | 0.47 | | GC | | 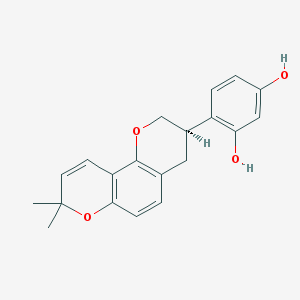 | |
| MOL004855 | | | | Licoricone | | 63.58 | | 0.47 | | GC | | 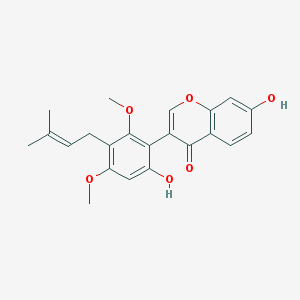 | |
| MOL004879 | | | | Glycyrin | | 52.61 | | 0.47 | | GC | | 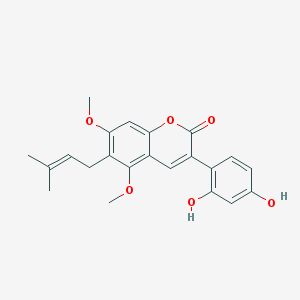 | |
| MOL005012 | | | | Licoagroisoflavone | | 57.28 | | 0.49 | | GC | | 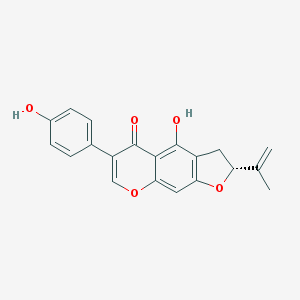 | |
| MOL004912 | | | | Glabrone | | 52.51 | | 0.5 | | GC | | 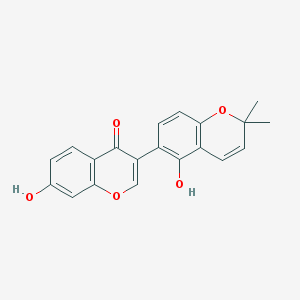 | |
| MOL004820 | | | | kanzonols W | | 50.48 | | 0.52 | | GC | | 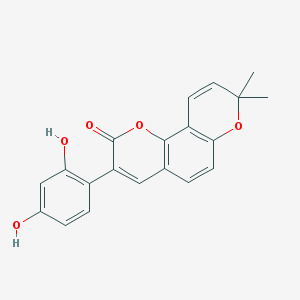 | |
| MOL004978 | | | | 2-[(3R)-8,8-dimethyl-3,4-dihydro-2H-pyrano[6,5-f]chromen-3-yl]-5-methoxyphenol | | 36.21 | | 0.52 | | GC | | 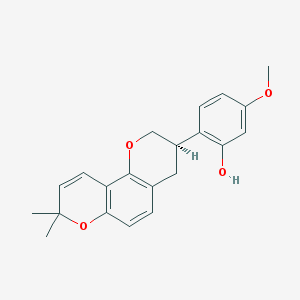 | |
| MOL004914 | | | | 1,3-dihydroxy-8,9-dimethoxy-6-benzofurano[3,2-c]chromenone | | 62.9 | | 0.53 | | GC | | 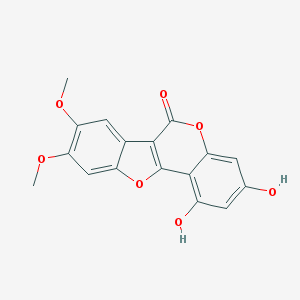 | |
| MOL004810 | | | | glyasperin F | | 75.84 | | 0.54 | | GC | | 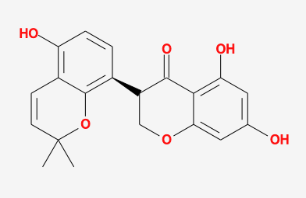 | |
| MOL001484 | | | | Inermine | | 75.18 | | 0.54 | | GC | | 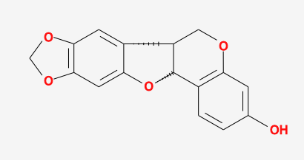 | |
| MOL004885 | | | | licoisoflavanone | | 52.47 | | 0.54 | | GC | |  | |
| MOL004884 | | | | Licoisoflavone B | | 38.93 | | 0.55 | | GC | |  | |
| MOL004827 | | | | Semilicoisoflavone B | | 48.78 | | 0.55 | | GC | |  | |
| MOL004806 | | | | euchrenone | | 30.29 | | 0.57 | | GC | |  | |
| MOL004974 | | | | 3'-Methoxyglabridin | | 46.16 | | 0.57 | | GC | |  | |
| MOL004966 | | | | 3'-Hydroxy-4'-O-Methylglabridin | | 43.71 | | 0.57 | | GC | |  | |
| MOL005017 | | | | Phaseol | | 78.77 | | 0.58 | | GC | |  | |
| MOL005003 | | | | Licoagrocarpin | | 58.81 | | 0.58 | | GC | |  | |
| MOL005007 | | | | Glyasperins M | | 72.67 | | 0.59 | | GC | |  | |
| MOL004824 | | | | (2S)-6-(2,4-dihydroxyphenyl)-2-(2-hydroxypropan-2-yl)-4-methoxy-2,3-dihydrofuro[3,2-g]chromen-7-one | | 60.25 | | 0.63 | | GC | |  | |
| MOL004959 | | | | 1-Methoxyphaseollidin | | 69.98 | | 0.64 | | GC | |  | |
| MOL004904 | | | | licopyranocoumarin | | 80.36 | | 0.65 | | GC | |  | |
| MOL002311 | | | | Glycyrol | | 90.78 | | 0.67 | | GC | |  | |
| MOL004805 | | | | (2S)-2-[4-hydroxy-3-(3-methylbut-2-enyl)phenyl]-8,8-dimethyl-2,3-dihydropyrano[2,3-f]chromen-4-one | | 31.79 | | 0.72 | | GC | |  | |
| MOL004891 | | | | shinpterocarpin | | 80.3 | | 0.73 | | GC | |  | |
| MOL005001 | | | | Gancaonin H | | 50.1 | | 0.78 | | GC | |  | |
| MOL004948 | | | | Isoglycyrol | | 44.7 | | 0.84 | | GC | |  | |
| MOL005018 | | | | Xambioona | | 54.85 | | 0.87 | | GC | |  | |
| MOL004988 | | | | Kanzonol F | | 32.47 | | 0.89 | | GC | |  | |
| MOL007514 | | | | methyl icosa-11,14-dienoate | | 39.67 | | 0.23 | | DS | |  | |
| MOL008400 | | | | glycitein | | 50.48 | | 0.24 | | DS | |  | |
| MOL002140 | | | | Perlolyrine | | 65.95 | | 0.27 | | DS | |  | |
| MOL005321 | | | | Frutinone A | | 65.9 | | 0.34 | | DS | |  | |
| MOL002879 | | | | Diop | | 43.59 | | 0.39 | | DS | |  | |
| MOL007059 | | | | 3-beta-Hydroxymethyllenetanshiquinone | | 32.16 | | 0.41 | | DS | |  | |
| MOL004492 | | | | Chrysanthemaxanthin | | 38.72 | | 0.58 | | DS | |  | |
| MOL008411 | | | | 11-Hydroxyrankinidine | | 40 | | 0.66 | | DS | |  | |
| MOL006774 | | | | stigmast-7-enol | | 37.42 | | 0.75 | | DS | |  | |
| MOL004355 | | | | Spinasterol | | 42.98 | | 0.76 | | DS | |  | |
| MOL001006 | | | | poriferasta-7,22E-dien-3beta-ol | | 42.98 | | 0.76 | | DS | |  | |
| MOL003036 | | | | ZINC03978781 | | 43.83 | | 0.76 | | DS | |  | |
| MOL008407 | | | | (8S,9S,10R,13R,14S,17R)-17-[(E,2R,5S)-5-ethyl-6-methylhept-3-en-2-yl]-10,13-dimethyl-1,2,4,7,8,9,11,12,14,15,16,17-dodecahydrocyclopenta[a]phenanthren-3-one | | 45.4 | | 0.76 | | DS | |  | |
| MOL006554 | | | | Taraxerol | | 38.4 | | 0.77 | | DS | |  | |
| MOL008397 | | | | Daturilin | | 50.37 | | 0.77 | | DS | |  | |
| MOL008391 | | | | 5alpha-Stigmastan-3,6-dione | | 33.12 | | 0.79 | | DS | |  | |
| MOL000072 | | | | 8β-ethoxy atractylenolide Ⅲ | | 35.95 | | 0.21 | | BZ | |  | |
| MOL000049 | | | | 3β-acetoxyatractylone | | 54.07 | | 0.22 | | BZ | |  | |
| MOL000020 | | | | 12-senecioyl-2E,8E,10E-atractylentriol | | 62.4 | | 0.22 | | BZ | |  | |
| MOL000022 | | | | 14-acetyl-12-senecioyl-2E,8Z,10E-atractylentriol | | 63.37 | | 0.3 | | BZ | |  | |
| MOL000021 | | | | 14-acetyl-12-senecioyl-2E,8E,10E-atractylentriol | | 60.31 | | 0.31 | | BZ | |  | |
| MOL000028 | | | | α-Amyrin | | 39.51 | | 0.76 | | BZ | |  | |
| MOL000033 | | | | (3S,8S,9S,10R,13R,14S,17R)-10,13-dimethyl-17-[(2R,5S)-5-propan-2-yloctan-2-yl]-2,3,4,7,8,9,11,12,14,15,16,17-dodecahydro-1H-cyclopenta[a]phenanthren-3-ol | | 36.23 | | 0.78 | | BZ | |  | |
| MOL000300 | | | | dehydroeburicoic acid | | 44.17 | | 0.83 | | FL | |  | |
| MOL000282 | | | | ergosta-7,22E-dien-3beta-ol | | 43.51 | | 0.72 | | FL | |  | |
| MOL000283 | | | | Ergosterol peroxide | | 40.36 | | 0.81 | | FL | |  | |
| MOL000275 | | | | trametenolic acid | | 38.71 | | 0.8 | | FL | |  | |
| MOL000287 | | | | 3beta-Hydroxy-24-methylene-8-lanostene-21-oic acid | | 38.7 | | 0.81 | | FL | |  | |
| MOL000285 | | | | (2R)-2-[(5R,10S,13R,14R,16R,17R)-16-hydroxy-3-keto-4,4,10,13,14-pentamethyl-1,2,5,6,12,15,16,17-octahydrocyclopenta[a]phenanthren-17-yl]-5-isopropyl-hex-5-enoic acid | | 38.26 | | 0.82 | | FL | |  | |
| MOL000292 | | | | poricoic acid C | | 38.15 | | 0.75 | | FL | |  | |
| MOL000279 | | | | Cerevisterol | | 37.96 | | 0.77 | | FL | |  | |
| MOL000296 | | | | hederagenin | | 36.91 | | 0.75 | | FL，TR | |  | |
| MOL000276 | | | | 7,9(11)-dehydropachymic acid | | 35.11 | | 0.81 | | FL | |  | |
| MOL000289 | | | | pachymic acid | | 33.63 | | 0.81 | | FL | |  | |
| MOL000280 | | | | (2R)-2-[(3S,5R,10S,13R,14R,16R,17R)-3,16-dihydroxy-4,4,10,13,14-pentamethyl-2,3,5,6,12,15,16,17-octahydro-1H-cyclopenta[a]phenanthren-17-yl]-5-isopropyl-hex-5-enoic acid | | 31.07 | | 0.82 | | FL | |  | |
| MOL000273 | | | | (2R)-2-[(3S,5R,10S,13R,14R,16R,17R)-3,16-dihydroxy-4,4,10,13,14-pentamethyl-2,3,5,6,12,15,16,17-octahydro-1H-cyclopenta[a]phenanthren-17-yl]-6-methylhept-5-enoic acid | | 30.93 | | 0.81 | | FL | |  | |
| MOL001323 | | | | Sitosterol alpha1 | | 43.28 | | 0.78 | | YYR，TR | |  | |
| MOL001494 | | | | Mandenol | | 42 | | 0.19 | | YYR | |  | |
| MOL002372 | | | | (6Z,10E,14E,18E)-2,6,10,15,19,23-hexamethyltetracosa-2,6,10,14,18,22-hexaene | | 33.55 | | 0.42 | | YYR | |  | |
| MOL002882 | | | | [(2R)-2,3-dihydroxypropyl] (Z)-octadec-9-enoate | | 34.13 | | 0.3 | | YYR | |  | |
| MOL008118 | | | | Coixenolide | | 32.4 | | 0.43 | | YYR | |  | |
| MOL008121 | | | | 2-Monoolein | | 34.23 | | 0.29 | | YYR | |  | |
| MOL000953 | | | | CLR | | 37.87 | | 0.68 | | YYR，BZL，LK，TBC | |  | |
| MOL007991 | | | | 2-methoxy-9,10-dihydrophenanthrene-4,5-diol | | 44.97 | | 0.18 | | SCG | |  | |
| MOL000173 | | | | wogonin | | 30.68 | | 0.23 | | BZL | |  | |
| MOL001735 | | | | Dinatin | | 30.97 | | 0.27 | | BZL | |  | |
| MOL002719 | | | | 6-Hydroxynaringenin | | 33.23 | | 0.24 | | BZL | |  | |
| MOL002714 | | | | baicalein | | 33.52 | | 0.21 | | BZL | |  | |
| MOL001755 | | | | 24-Ethylcholest-4-en-3-one | | 36.08 | | 0.76 | | BZL | |  | |
| MOL012245 | | | | 5,7,4'-trihydroxy-6-methoxyflavanone | | 36.63 | | 0.27 | | BZL | |  | |
| MOL005869 | | | | daucostero_qt | | 36.91 | | 0.75 | | BZL | |  | |
| MOL012251 | | | | Chrysin-5-methylether | | 37.27 | | 0.2 | | BZL | |  | |
| MOL012254 | | | | campesterol | | 37.58 | | 0.71 | | BZL | |  | |
| MOL012266 | | | | rivularin | | 37.94 | | 0.37 | | BZL | |  | |
| MOL012252 | | | | 9,19-cyclolanost-24-en-3-ol | | 38.69 | | 0.78 | | BZL | |  | |
| MOL001973 | | | | Sitosteryl acetate | | 40.39 | | 0.85 | | BZL | |  | |
| MOL001040 | | | | (2R)-5,7-dihydroxy-2-(4-hydroxyphenyl)chroman-4-one | | 42.36 | | 0.21 | | BZL | |  | |
| MOL012250 | | | | 7-hydroxy-5,8-dimethoxy-2-phenyl-chromone | | 43.72 | | 0.25 | | BZL | |  | |
| MOL008206 | | | | Moslosooflavone | | 44.09 | | 0.25 | | BZL | |  | |
| MOL012270 | | | | Stigmastan-3,5,22-triene | | 45.03 | | 0.71 | | BZL | |  | |
| MOL012269 | | | | Stigmasta-5,22-dien-3-ol-acetate | | 46.44 | | 0.86 | | BZL | |  | |
| MOL000351 | | | | Rhamnazin | | 47.14 | | 0.34 | | BZL | |  | |
| MOL002915 | | | | Salvigenin | | 49.07 | | 0.33 | | BZL | |  | |
| MOL012248 | | | | 5-hydroxy-7,8-dimethoxy-2-(4-methoxyphenyl)chromone | | 65.82 | | 0.33 | | BZL | |  | |
| MOL005190 | | | | eriodictyol | | 71.79 | | 0.24 | | BZL | |  | |
| MOL012246 | | | | 5,7,4'-trihydroxy-8-methoxyflavanone | | 74.24 | | 0.26 | | BZL | |  | |
| MOL002058 | | | | 40957-99-1 | | 57.2 | | 0.62 | | LK | |  | |
| MOL002773 | | | | beta-carotene | | 37.18 | | 0.58 | | LK | |  | |
| MOL000546 | | | | diosgenin | | 80.88 | | 0.81 | | LK | |  | |
| MOL007356 | | | | solanocapsine | | 52.94 | | 0.67 | | LK | |  | |
| MOL001646 | | | | 2,3-dimethoxy-6-methyanthraquinone | | 34.86 | | 0.26 | | BHSSC | |  | |
| MOL001659 | | | | Poriferasterol | | 43.83 | | 0.76 | | BHSSC | |  | |
| MOL001663 | | | | (4aS,6aR,6aS,6bR,8aR,10R,12aR,14bS)-10-hydroxy-2,2,6a,6b,9,9,12a-heptamethyl-1,3,4,5,6,6a,7,8,8a,10,11,12,13,14b-tetradecahydropicene-4a-carboxylic acid | | 32.03 | | 0.76 | | BHSSC | |  | |
| MOL001670 | | | | 2-methoxy-3-methyl-9,10-anthraquinone | | 37.83 | | 0.21 | | BHSSC | |  | |
| MOL000493 | | | | campesterol | | 37.58 | | 0.71 | | TR | |  | |
| MOL001340 | | | | GA120 | | 84.85 | | 0.45 | | TR | |  | |
| MOL001371 | | | | Populoside_qt | | 108.89 | | 0.2 | | TR | |  | |
